# Supplementary material for: Structure-based design of a phosphotyrosine-masked covalent ligand targeting the E3 ligase SOCS2
Source: Nat Commun. 2023 Oct 10;14:6345. doi: 10.1038/s41467-023-41894-3 (PMC10564737; doi:10.1038/s41467-023-41894-3)
Supplement: Supplementary file 1 — Supplementary Information [file 41467_2023_41894_MOESM1_ESM.pdf]

Supporting information for:

Structure-based design of a phosphotyrosine-masked covalent ligand targeting the E3 ligase SOCS2

Sarath Ramachandran[1,4], Nikolai Makukhin[1,3,4], Kevin Haubrich[1], Manjula Nagala[1], Beth Forrester[1], Dylan M. Lynch[1], Ryan Casement[1], Andrea Testa[1,3], Elvira Bruno[1], Rosaria Gitto[2], Alessio Ciulli [1,*]

Affiliations:

[1] Centre for Targeted Protein Degradation, Division of Biological Chemistry and Drug Discovery, School of Life Sciences, University of Dundee, 1 James Lindsay Place, Dundee, DD1 5JJ, United Kingdom

[2] Department of Chemical, Biological, Pharmaceutical, and Environmental Sciences, University of Messina, Viale Stagno D'Alcontres 31, Pole Papardo, 98166, Messina, Italy

[3] Present address: Amphista Therapeutics Ltd, Cory Building, Granta Park, Great Abington, Cambridge, CB21 6GQ, United Kingdom

[4] These authors contributed equally

Correspondence and requests for materials should be addressed to A.C. (email: a.ciulli@dundee.ac.uk)

Table of Contents

Structure-based design of a phosphotyrosine-masked covalent ligand targeting the E3 ligase SOCS2 . 1

Supplementary Figure 1. Solid-phase synthesis of *N*-terminally modified *N*-methylcarboxamide phosphotyrosines. 3

Supplementary Figure 2. Synthesis of the second-round library of SOCS2 ligands. 3

Supplementary Figure 3. Synthesis of MN551 3

Supplementary Figure 4. Synthesis of MN551 prodrugs 3

Supplementary Figure 5: NMR spectra of MN714 and MN551 4

Supplementary Figure 6: Electron density maps for SBC binders. 6

Supplementary Figure 7: SPR sensograms obtained for the SBC binders. 7

Supplementary Figure 8: ITC titration curves obtained for the SBC binders. 9

Supplementary Figure 9: A representative FP assay cascade used to determine the kinetic parameters defining the covalent efficiency of the MN551. 10

Supplementary Figure 10: GSH kinetic study of compound MN551 in GSH stability assay. 11

Supplementary Figure 11: Cell viability assay to assess cytotoxicity of MN714. 11

Supplementary Figure 12: Cycloheximide chase assay used to determine the steady state stability of SOCS2. (n=1) 12

Values in parentheses correspond to the highest resolution shell 13

SUPPLEMENTARY METHODS 14

Chemistry 14

REFERENCES 25

Supplementary Figure 1. Solid-phase synthesis of *N*-terminally modified *N*-methylcarboxamide phosphotyrosines.

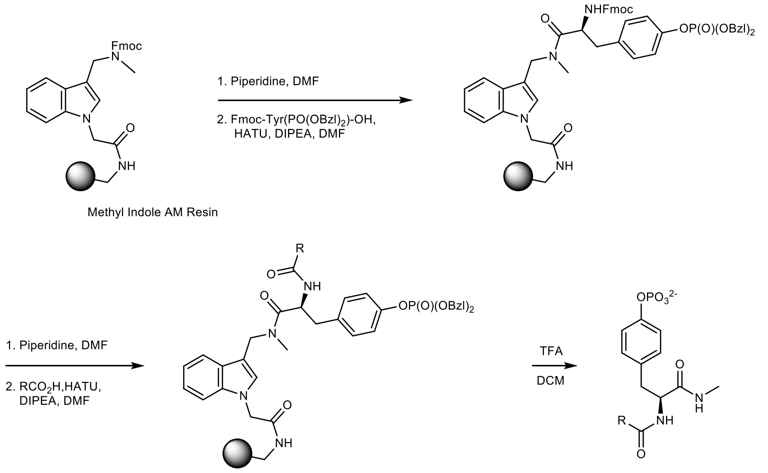

Supplementary Figure 2. Synthesis of the second-round library of SOCS2 ligands.

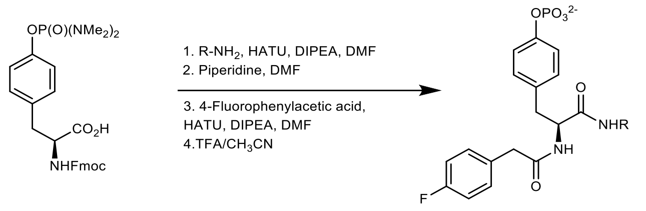

Supplementary Figure 3. Synthesis of MN551

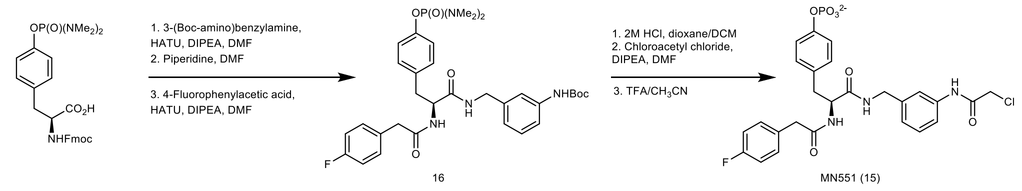

Supplementary Figure 4. Synthesis of MN551 prodrugs

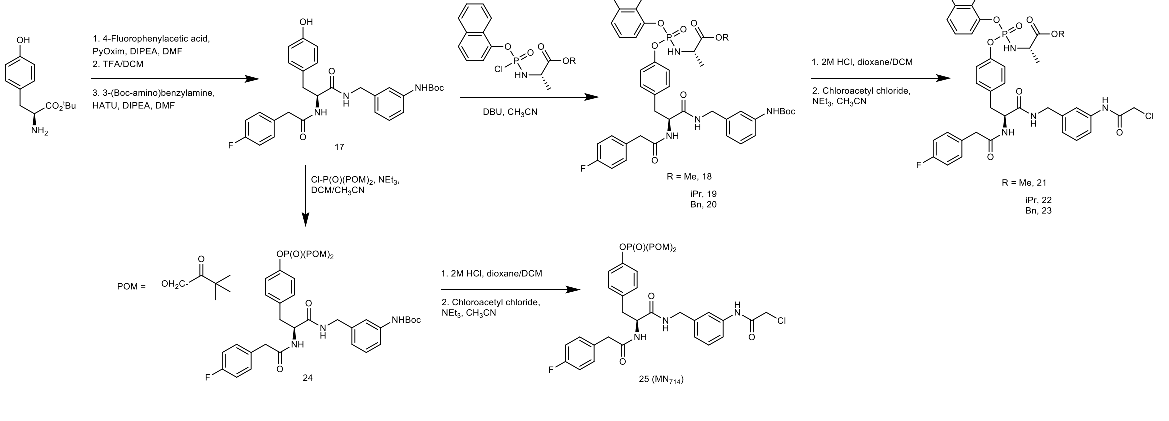

Supplementary Figure 5: NMR spectra of MN714 and MN551

MN714

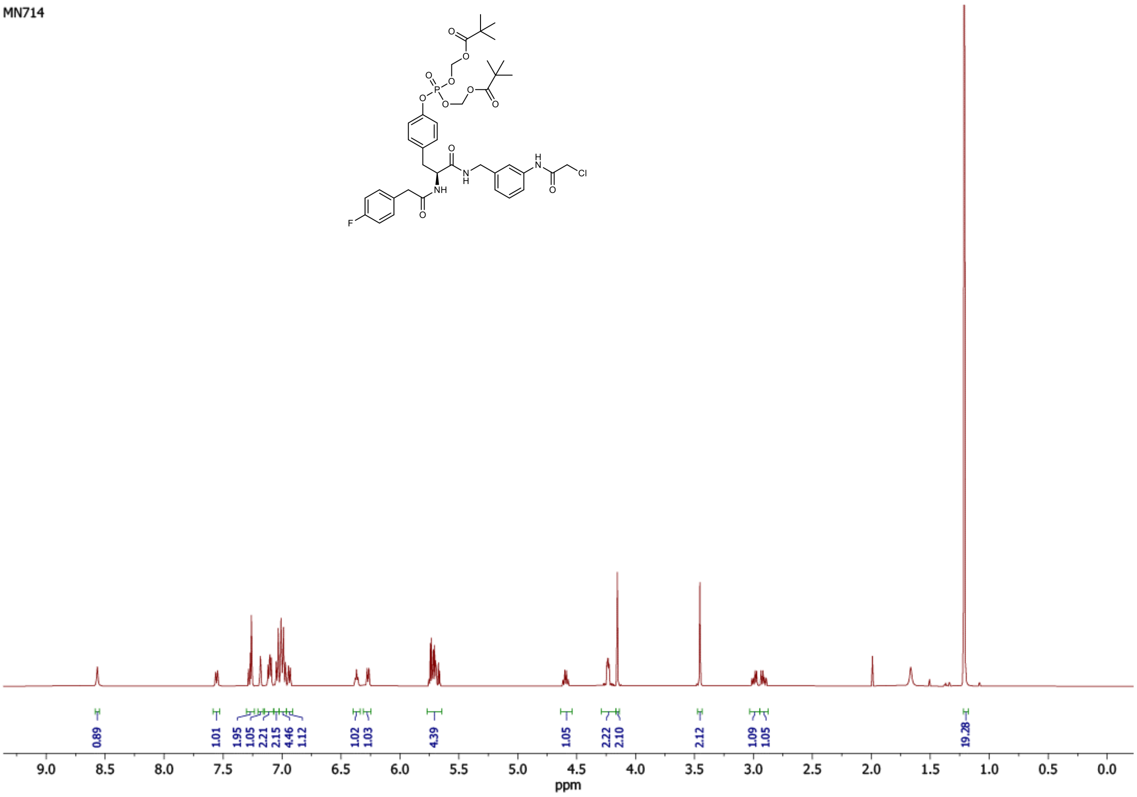

MN714

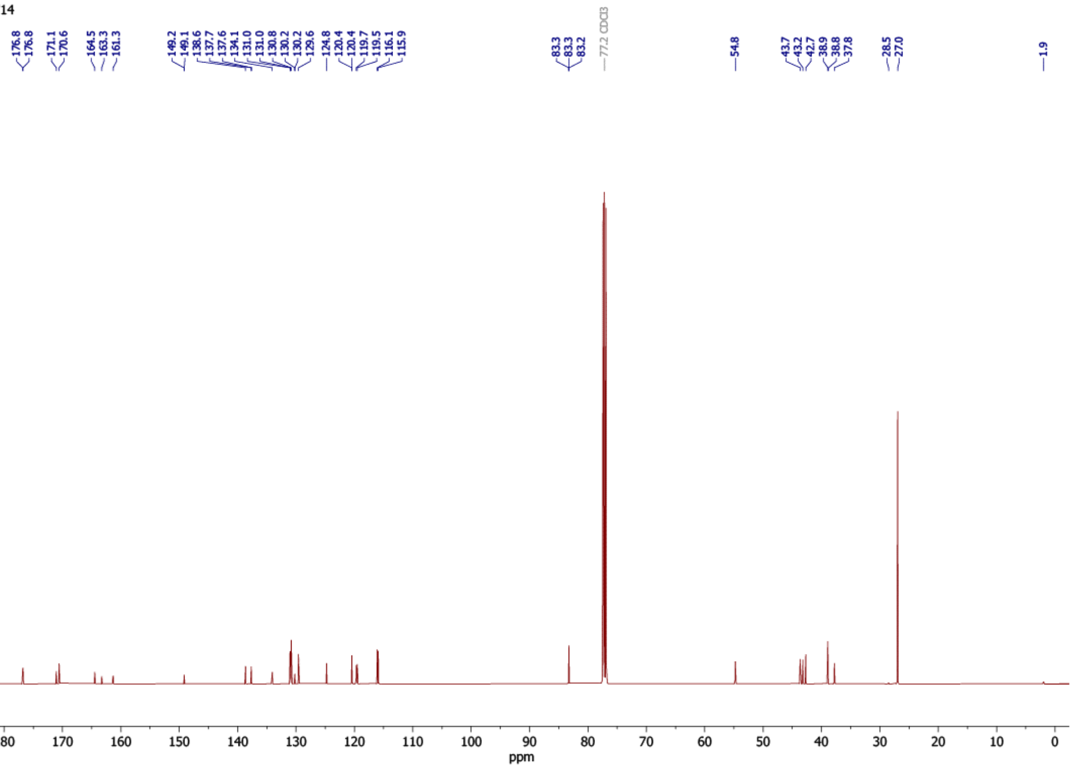

MN551

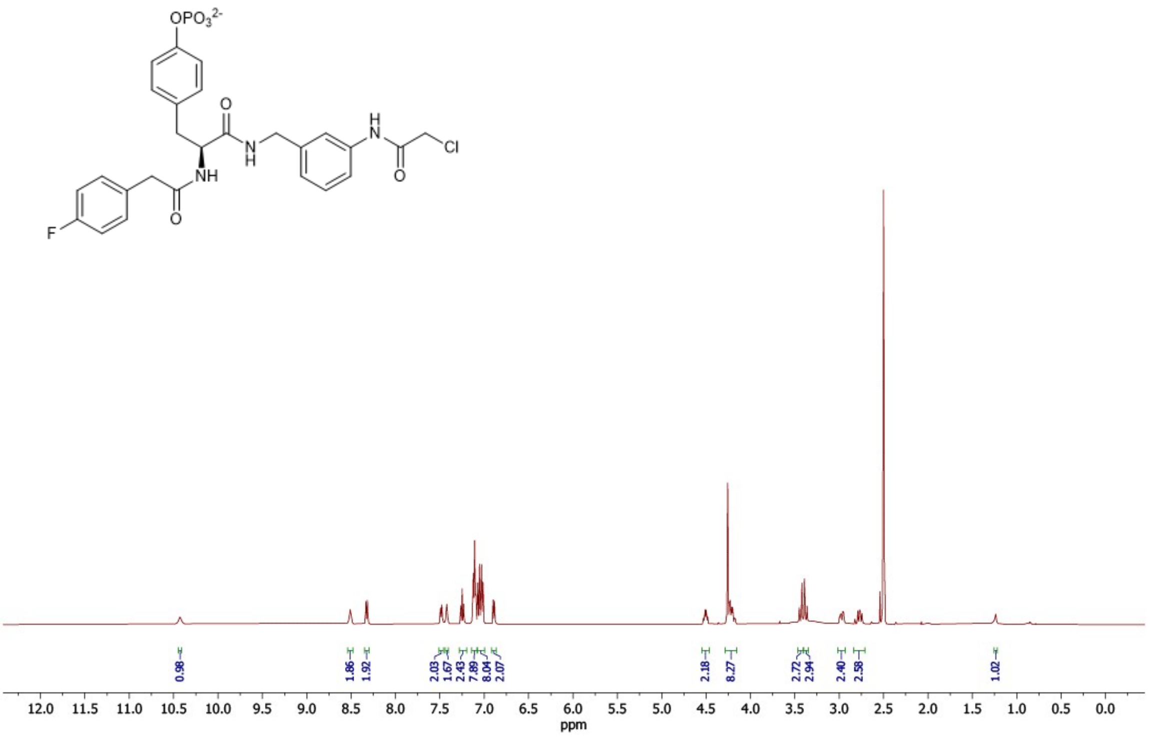
OPO3 2-
O
H
N
Cl
O
N
H
NH
O
F
0.98
1.86
1.92
2.03
1.67
2.43
7.89
8.04
2.07
2.18
8.27
2.72
2.94
2.40
2.58
1.02
12.0 11.5 11.0 10.5 10.0 9.5 9.0 8.5 8.0 7.5 7.0 6.5 6.0 5.5 5.0 4.5 4.0 3.5 3.0 2.5 2.0 1.5 1.0 0.5 0.0
ppm

MN551

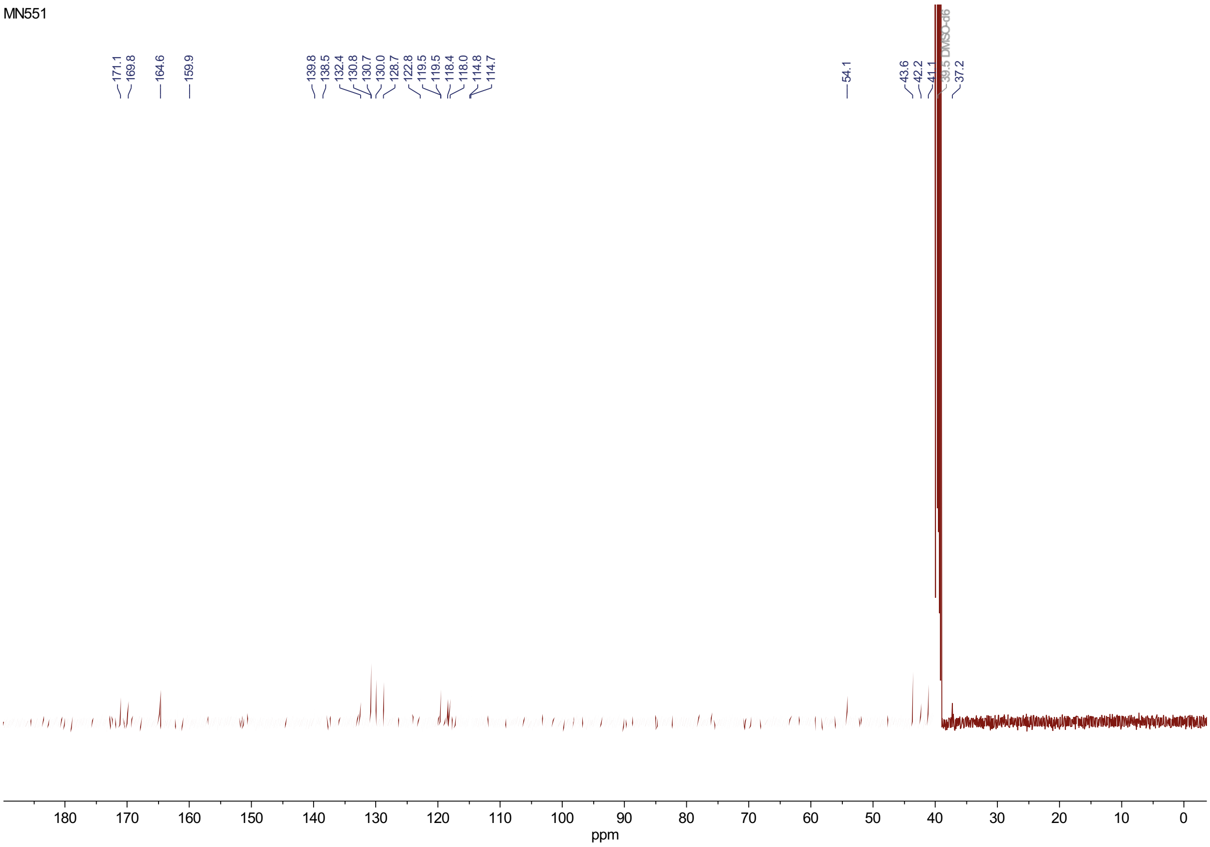
171.1
169.8
164.6
159.9
139.8
138.5
132.4
130.8
130.7
130.0
128.7
122.8
119.5
119.5
118.4
118.0
114.8
114.7
54.1
43.6
42.2
41.1
39.5 DMSO-d6
37.2
180 170 160 150 140 130 120 110 100 90 80 70 60 50 40 30 20 10 0
ppm

Supplementary Figure 6: Electron density maps for SBC binders. 2Fo-Fc map contoured at 1.0 σ level and Omit map contoured at 3.0 σ level

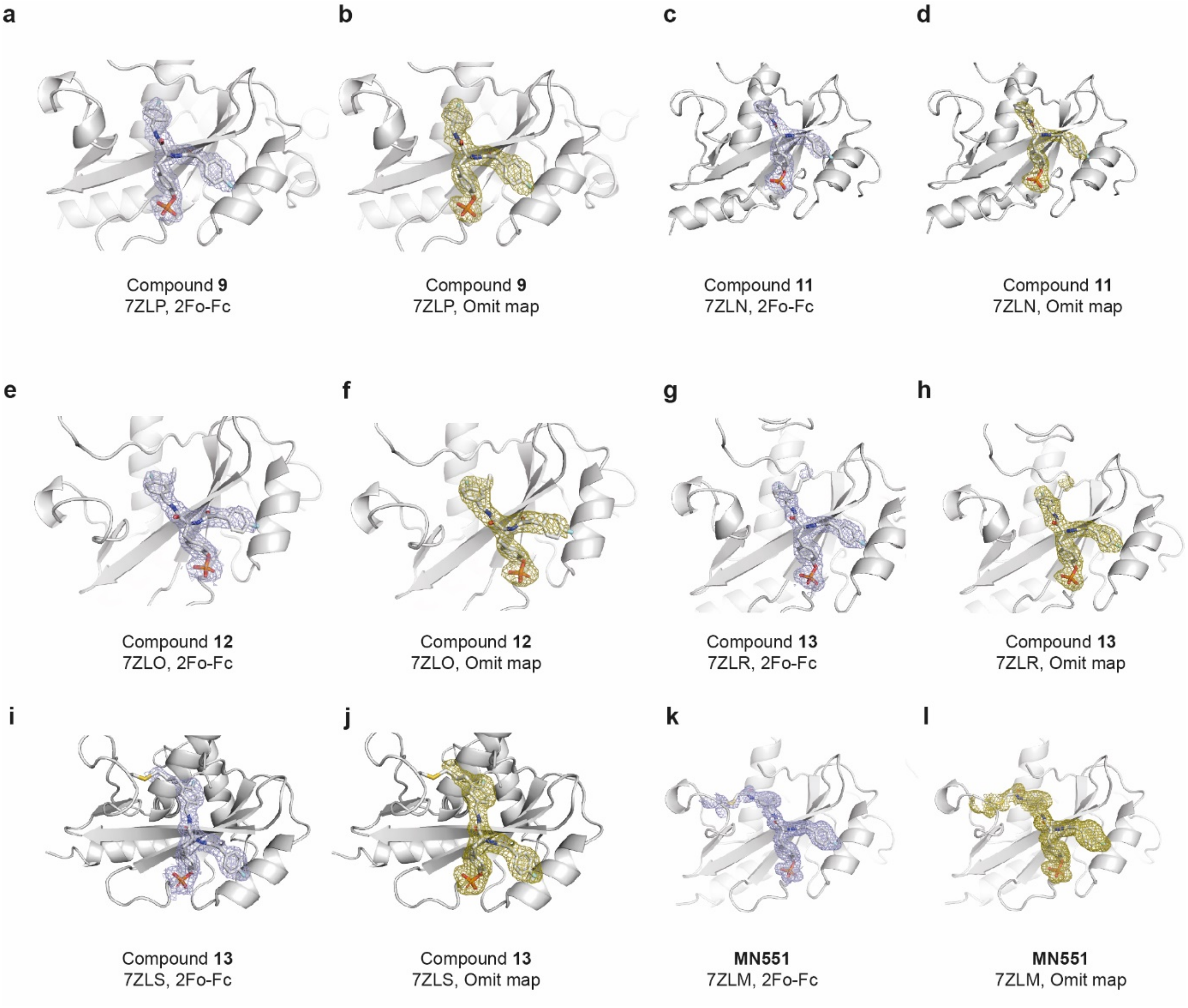

Supplementary Figure 7: SPR sensograms obtained for the SBC binders.

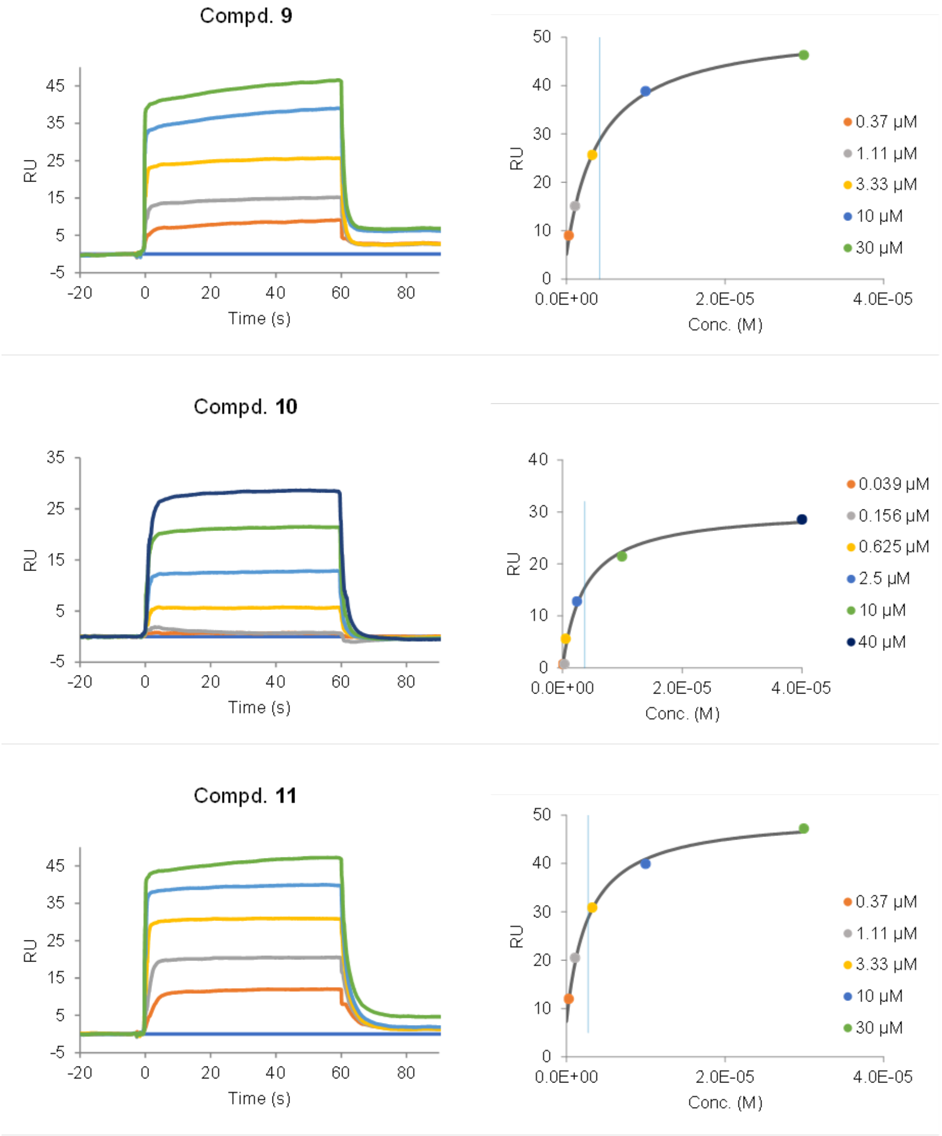

Compd. **12**

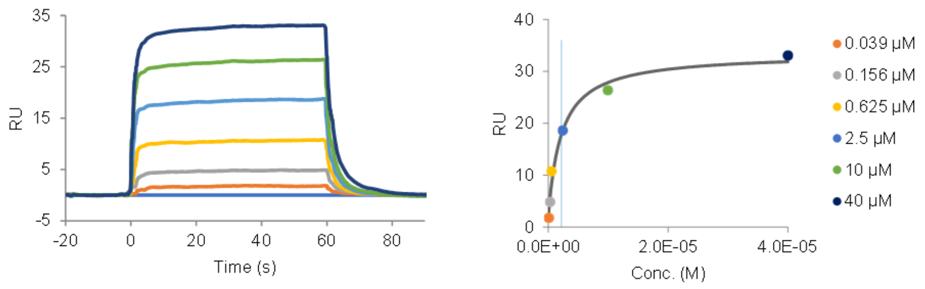

Compd. **13**

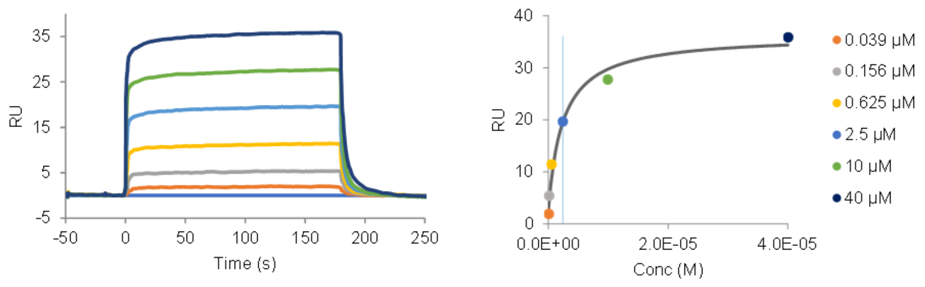

Compd. **14**

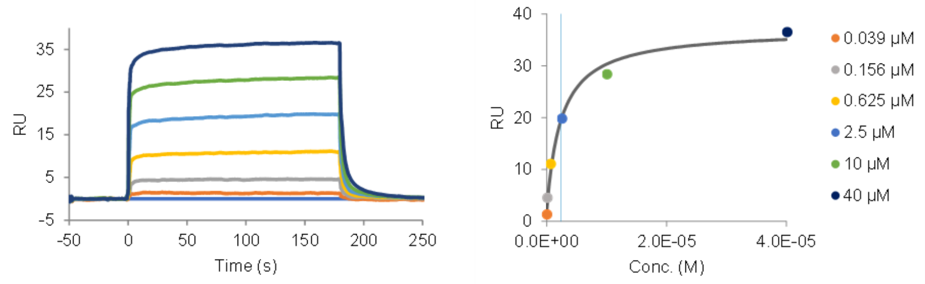

Supplementary Figure 8: ITC titration curves obtained for the SBC binders.

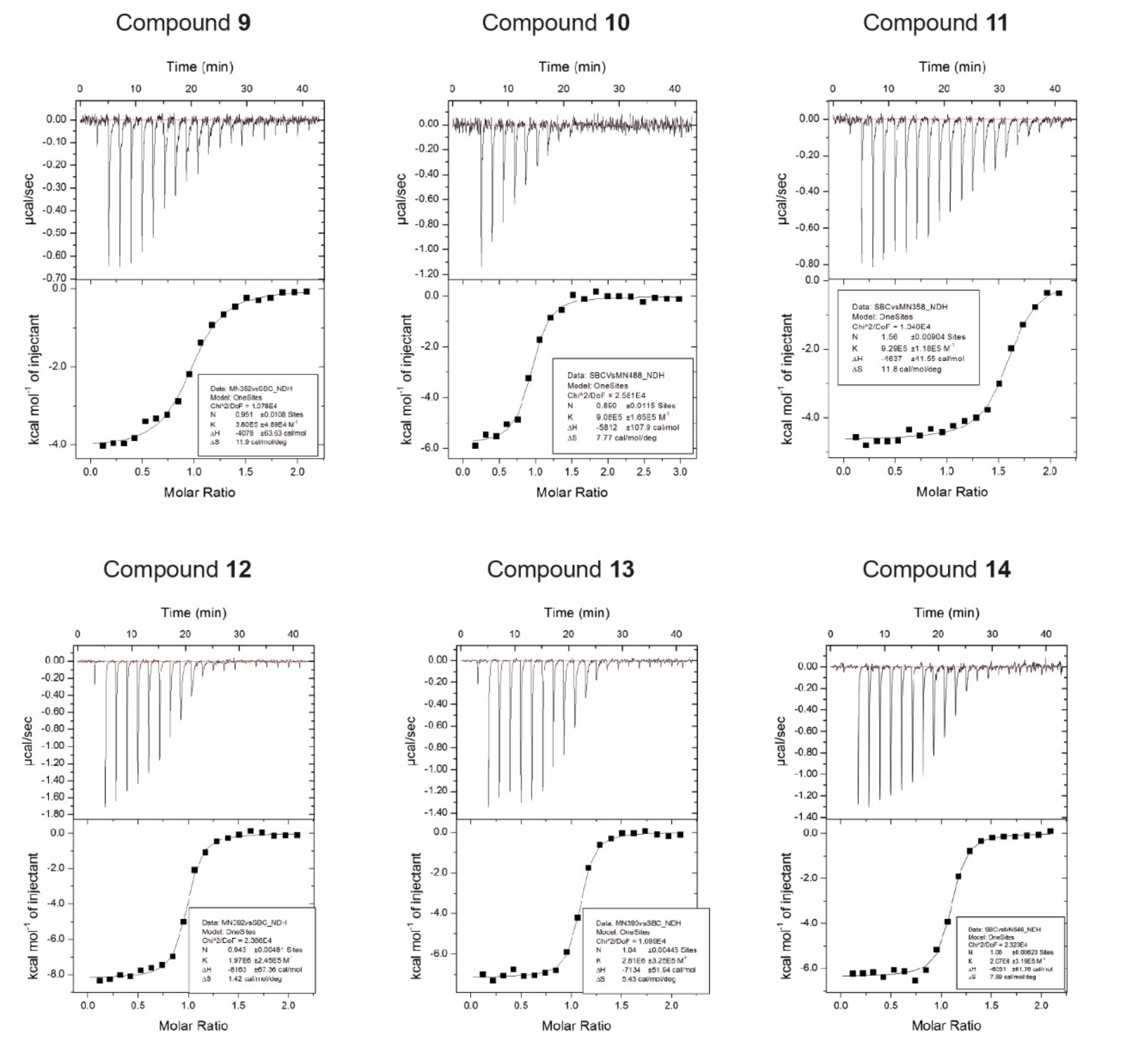

Supplementary Figure 9: A representative FP assay cascade used to determine the kinetic parameters defining the covalent efficiency of the MN551. a A plot of SBC titration with 10nM probe yielding K_d of the probe. **b** A representative plot of bound fraction of probe F_b v/s time obtained with competition from multiple doses of MN551. Data are presented as means±SD, from technical triplicates. **c** A representative plot of initial bound fraction F_{bo} v/s concentration of MN551. **d** A representative plot of k_{obs} v/s concentration of MN551 used to determine covalent parameters. **e** A representative plot of bound fraction of probe F_b v/s time obtained with competition from multiple doses of MN551 with concentrations less than K_i. Data are presented as means±SD, from technical triplicates. **f** A representative plot of k_{obs} v/s concentration of MN551. Slope of the curve gives the covalent efficiency k_{inact}/K_I used to determine covalent parameters (Value reported in 7A, 7C, 7D and 7F reported as mean±SEM, n=3).

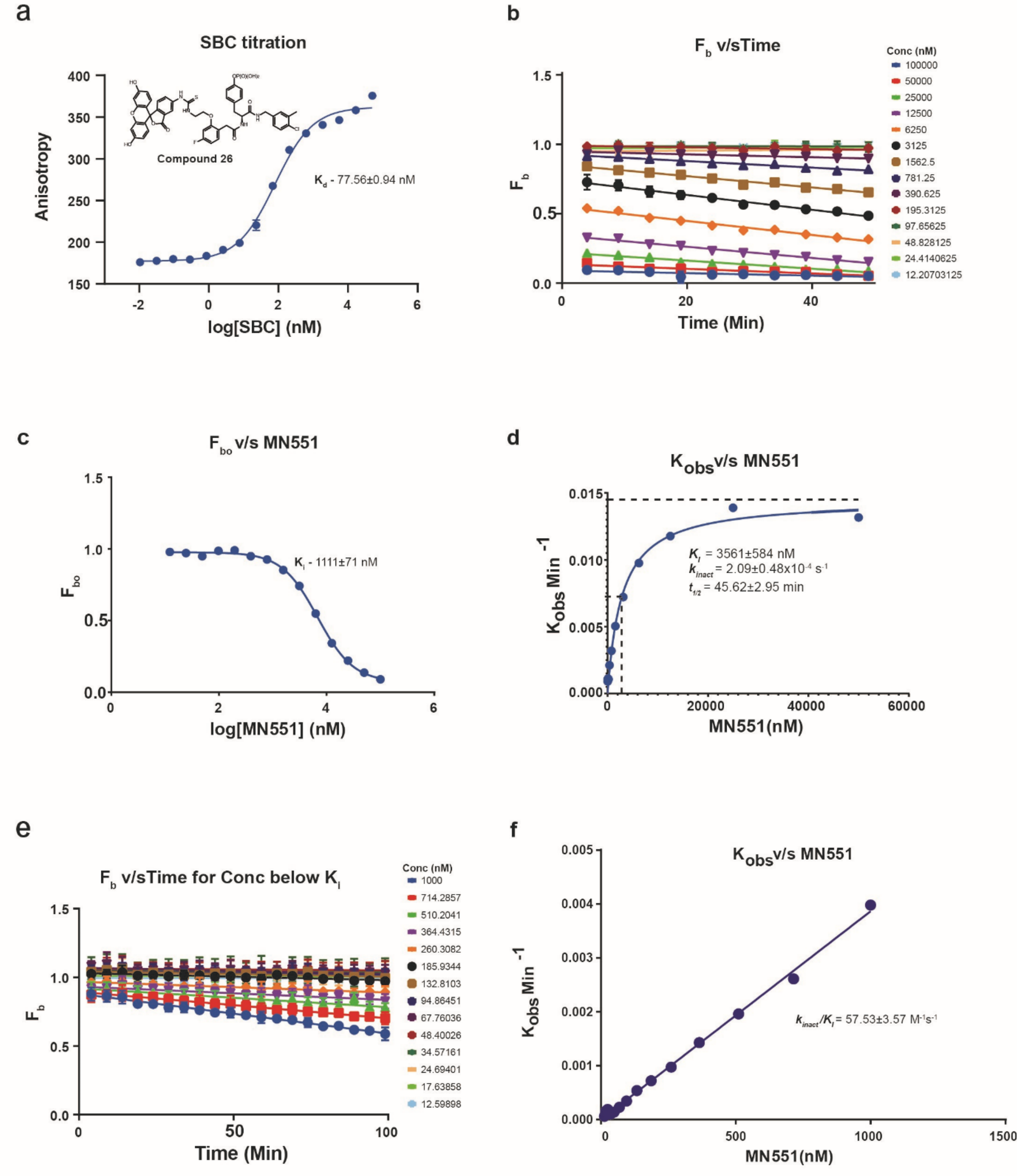

Supplementary Figure 10: GSH kinetic study of compound MN551 in GSH stability assay. A and **B**. A plot of unmodified **MN551** v/s time. The starting concentrations of **MN551** were 1 and 10 µM in **A** and **B** respectively. **C**. Reactivity of **MN551** measured against Afatinib and Ibrutinib

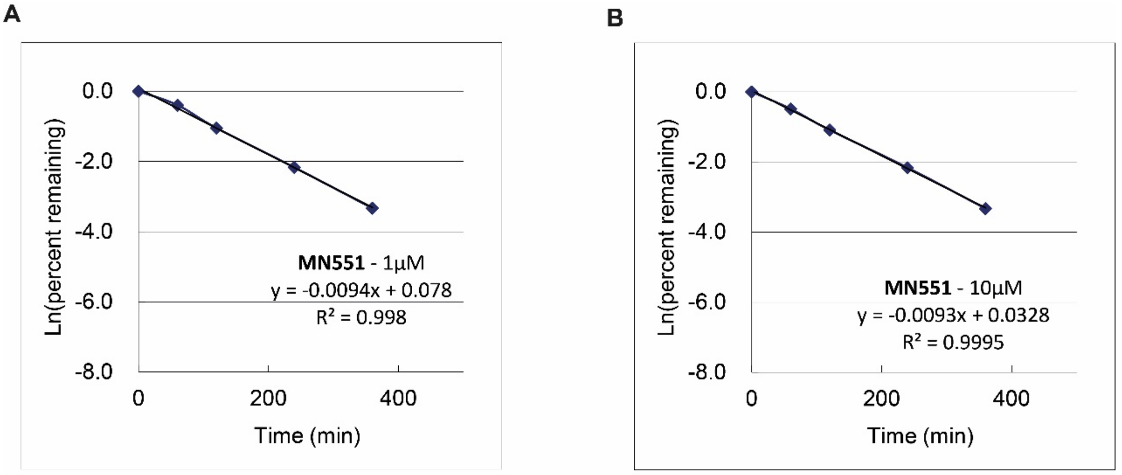

C

Sample Name	GSH Reaction Assay				
	R^2	$T_{1/2}$ (min)	Remaining (T=1440min)	Remaining (*NGSH=1440min)	Glutathione conjugation(Aa at T1440)
MN551-1um	0.9980	73.7	0.0%	112.9%	0
MN551-10um	0.9995	74.7	0.2%	101.1%	20753
Afatinib	0.9987	18.8	0.6%	107.0%	4181167
Ibrutinib	0.9988	384.8	7.9%	109.1%	192707

Supplementary Figure 11: Cell viability assay to assess cytotoxicity of MN714. (EC50 reported as mean±SEM, technical replicates=3).

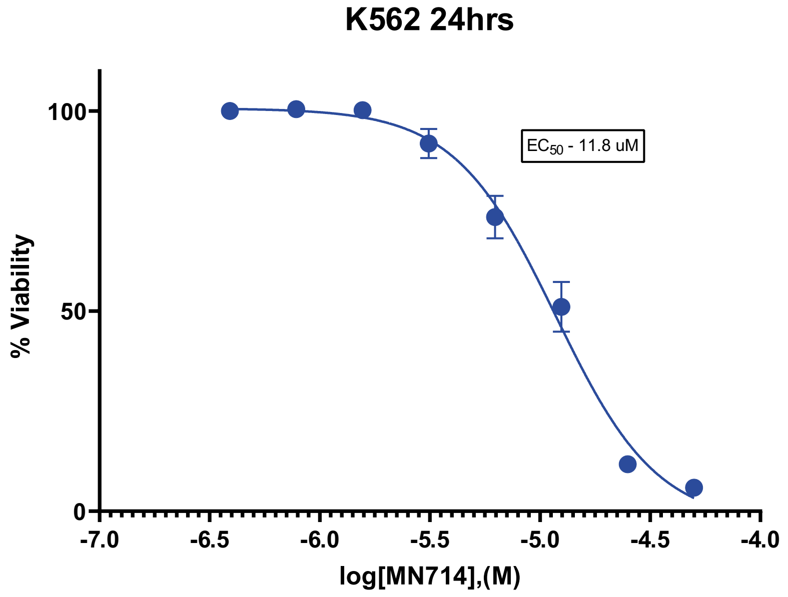

Supplementary Figure 12: Cyclohexamide chase assay used to determine the steady state stability of SOCS2. (n=1)

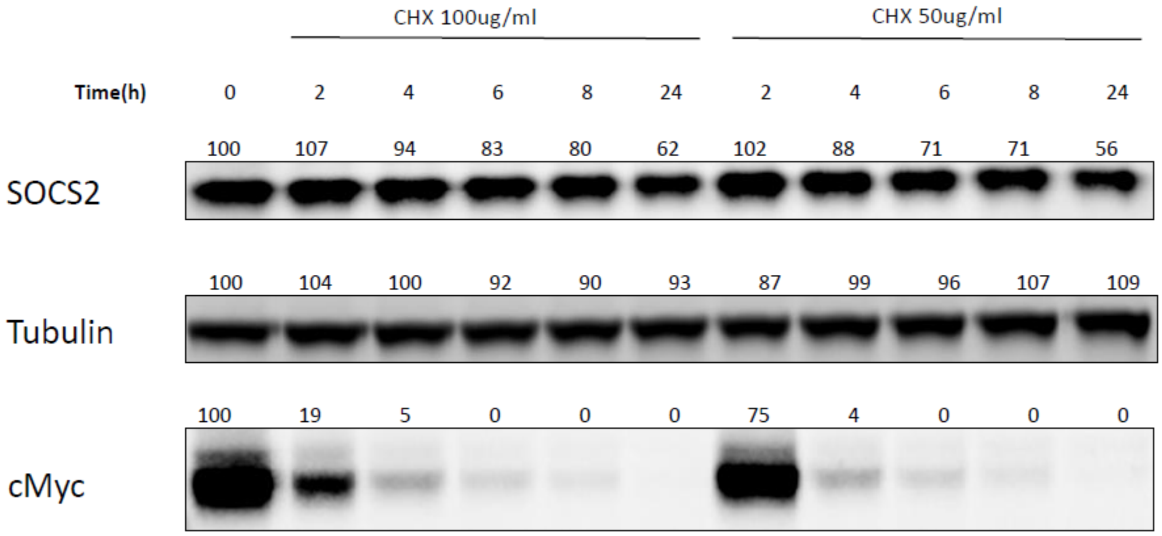

Supplementary Table 1: Crystallography table - data collection & refinement statistics.
Values in parentheses correspond to the highest resolution shell.

SBC complex	**9**	**11**	**12**	**13**	**13**	**MN551 (15)**
**Type**	Soak	Soak	Soak	Soak	Co-crystal	Co-crystal
**PDB code**	7ZLP	7ZLN	7ZLO	7ZLR	7ZLS	7ZLM
Data collection						
Wavelength (Å)	1.937	2.597	2.218	2.007	1.92	1.79
Space group	P 1 21 1	P 1 21 1	P 1 21 1	P 1 21 1	P 21 21 21	P 21 21 21
Cell dimensions						
a, b, c (Å)	56.1531, 52.2817, 77.453	56.6871, 52.258, 77.4912	55.926, 52.363, 77.583	56.112, 52.246, 77.458	59.5487, 172.641, 185.475	59.7846, 172.879, 185.809
α, β, γ (°)	90.00, 108.447, 90.00	90.00, 107.941, 90.00	90.00, 108.288, 90.00	90.00, 108.269, 90.00	90.00, 90.00, 90.00	90.00, 90.00, 90.00
Molecules/ASU	1	1	1	1	4	4
Resolution	42.6–1.937 (2.006–1.937)	53.93-2.597 (2.69-2.597)	37.81 - 2.218 (2.298 - 2.218)	53.28 - 2.007 (2.079 - 2.007)	63.18 - 1.92 (1.989 - 1.92)	63.28 - 1.79 (1.854- 1.79)
R_{merge} (%)	7.41 (72.89)	0.4353 (42.52)	4.417 (49.04)	5.418 (73.03)	2.722 (84.41)	4.81 (68.45)
<I/σ(I)>	5.67 (1.21)	3.02 (0.44)	8.56(1.4)	7.53(1.03)	13.78(0.44)	7.13(1.08)
Completeness (%)	99.69 (97.78)	94.33 (73.40)	92.31 (63.41)	85.77 (42.92)	98.44 (84.94)	99.61 (99.21)
Redundancy	2 (2)	2 (1.9)	2 (1.9)	2 (1.9)	1.9 (1.9)	2.0 (2.0)
$CC_{1/2}$	0.991 (0.162)	0.162 (0.00182)	0.997 (0.549	0.997 (0.307)	0.999 (0.428)	0.996 (0.432)
Refinement						
Resolution (Å)	1.94	2.60	2.22	2.07	1.92	1.79
Unique reflections	31815 (3080)	12762 (985)	19721 (1338)	24620 (1219)	146646 (14493)	181295 (17872)
R_{work}/R_{free} (%)	19.20/23.12	27.96/33.04	20.73/23.56	20.08/24.38	19.75/23.38	18.74/21.42
Wilson B factor (Å^2)	32.89	60.98	40.67	32.22	42.80	29.51
Average B factor (Å^2)	39.71	71.60	51.92	44.58	56.24	37.40
No. non-hydrogen atoms Protein/ligand/water	3074/40/183	2629/36/33	2795/37/81	2841/38/127	10757/152/567	10798/152/936
R.M.S.D.						
Bond lengths (Å)	0.007	0.003	0.002	0.002	0.007	0.008
Bond angles (°)	0.83	0.54	0.48	0.55	0.85	0.88
Ramachandran analysis						

Preferred regions (%)	98.02	96.51	97.44	97.74	96.35	97.15
Allowed regions (%)	1.98	3.49	2.46	2.26	3.65	2.85
Outliers (%)	0.0	0.0	0.0	0.0	0.0	0.0

SUPPLEMENTARY METHODS

Chemistry

All chemicals unless otherwise stated, were commercially available and used without further purification. Commercially available dry solvents were used. Flash column chromatography and reversed phase chromatography were performed using a Teledyne Isco Combiflash Rf with prepacked Redisep RF Normal phase disposable columns or RediSep Rf Gold C18 Reversed Phase columns, 20–40 microns. NMR Spectra were recorded on a Bruker 400 MHz or 500 MHz as specified. ^{13}C, ^{31}P, ^{19}F spectra were ^{1}H decoupled. Chemical shifts (δ) are reported in ppm and referenced to the residual solvent signals: ^{1}H NMR δ (ppm) = 7.26 ($CDCl_3$), ^{13}C NMR δ (ppm) = 77 ($CDCl_3$); ^{1}H NMR δ (ppm) = 2.50 (DMSO-d_6), ^{13}C NMR δ (ppm) = 39.50 (DMSO-d_6); ^{1}H NMR δ (ppm) = 3.31 (CD_3OD), ^{13}C NMR δ (ppm) = 49.00 (CD_3OD). Signal splitting patterns are described as singlet (s), doublet (d), triplet (t), quartet (q), multiplet (m), broad (br) or a combination thereof. Coupling constants (*J*) are measured in Hertz (Hz). High Resolution Mass Spectra (HRMS) were recorded on a Bruker microTOF. Other resolution MS and analytical HPLC traces were recorded on an Agilent Technologies 1200 series HPLC connected to an Agilent Technologies 6130 quadrupole LC/MS, connected to an Agilent diode array detector. The column used was a Waters XBridge column (50 mm × 2.1 mm, 3.5 µm particle size) and the compounds were eluted with a gradient 5−95% acetonitrile/water + 0.1 formic acid ("acidic method"). HPLC purification was performed on a Gilson Preparative HPLC System with a Waters XBridge C18 column (100 mm x 19 mm; 5 µm particle size) and a gradient of 5% to 95% acetonitrile in water over 10 min, flow 25 mL/min, with 0.1% formic acid or ammonia in the aqueous phase. cLogP calculations were performed in StarDrop v7.3.1.33573.

Solid-phase synthesis of N-terminally modified N-methylcarboxamide phosphotyrosines

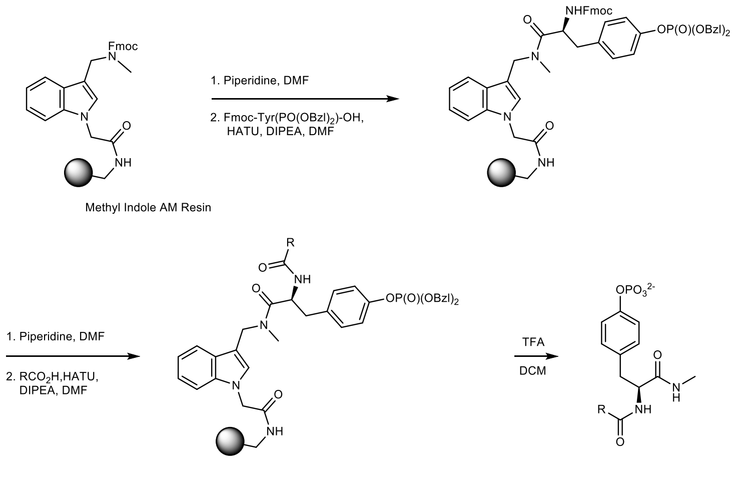

Compounds 1-8 were prepared via solid-phase peptide synthesis on 0.033 mmol scale using standard Fmoc chemistry on Methyl Indole AM resin (0.67 mmol/g) on an INTAVIS ResPepSL automated peptide synthesizer. O-(dibenzylphosphono)-N-Fmoc-L-tyrosine was synthesized as described previously[1]. Phosphotyrosines were cleaved with a mixture (1 mL) of TFA/DCM =1/1. The crude products were

purified by preparative HPLC under acidic conditions, UV detection at λ_{obs} = 190 and 210 nm, to give the desired compounds.

(*S*)-4-(2-acetamido-3-(methylamino)-3-oxopropyl)phenyl phosphate (1)

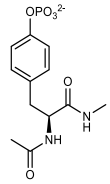

Compound **1** was obtained in 38% yield (4 mg). ^{1}H NMR (500 MHz, CD_3OD): 7.22 (2H, d, *J* = 8.0 Hz), 7.08 (2H, d, *J* = 8.0 Hz), 4.5 (1H, m), 3.07 (1H, dd, *J* = 6.4, 13.8 Hz), 2.85 (1H, dd, *J* = 8.7, 13.8 Hz), 2.67 (3H, s). 1.9 (3H, s). ^{13}C NMR (125 MHz, CD_3OD): 174.0, 173.1, 151.2, 151.2, 135.1, 131.5, 121.3, 121.2, 56.3, 38.3, 26.3, 22.4. MS (ESI) for $C_{12}H_{17}FN_2O_6P$ [M + H^+] calculated 317.1, obtained 317.1.

(*S*)-4-(2-(2-cyclopropylacetamido)-3-(methylamino)-3-oxopropyl)phenyl phosphate (2)

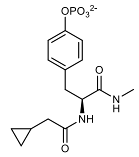

Compound **2** was obtained in 50% yield (5.8 mg). ^{1}H NMR (500 MHz, CD_3OD): 8.06 (1H ,s), 7.21 (2H, d, *J* = 8.5 Hz), 7.12 (2H, d, *J* = 8.5 Hz), 4.55 (1H, dd, *J* = 6.1, 8.8 Hz), 3.09 (1H, dd, *J* = 6.1, 13.9 Hz), 2.85 (1H, dd, *J* = 8.8, 13.9 Hz), 2.69 (3H, s), 2.07 (2H, m), 0.89 (1H, m), 0.46 (2H, m), 0.10 (2H, m). ^{13}C NMR (125 MHz, CD_3OD): 175.5, 174.0, 164.4, 163.3, 151.8, 151.7, 134.8, 131.4, 121.3, 121.2, 56.0, 41.8, 38.4, 26.3, 8.4, 4.9, 4.8. ^{31}P NMR (CD_3OD): -3.7. HRMS (ESI) for $C_{15}H_{22}N_2O_6P$ [M + H^+] calculated 357.1215, obtained 357.1207.

(*S*)-4-(3-(methylamino)-2-(2-morpholinoacetamido)-3-oxopropyl)phenyl phosphate (3)

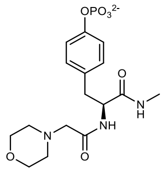

Compound **2** was obtained in 30% yield (4.0 mg). ^{1}H NMR (500 MHz, CD_3OD): 7.24 (2H, d, *J* = 8.5 Hz), 7.13 (2H, d, *J* = 8.5 Hz), 4.66 (1H, dd, *J* = 5.5, 9.8 Hz), 4.05-3.95 (3H, m), 3.88-3.68 (3H, m), 3.45 (1H, m), 3.24-3.12 (3H, m), 3.06 (1H, m), 2.83 (1H, m), 2.71 (3H, s). ^{31}P NMR (CD_3OD): -3.7. MS (ESI) for $C_{16}H_{25}N_3O_7P$ [M + H^+] calculated 402.1, obtained 402.1.

(*S*)-4-(3-(methylamino)-3-oxo-2-(2-phenylacetamido)propyl)phenyl phosphate (4)

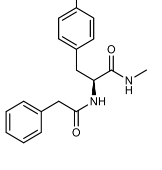

Compound **4** was obtained in 64% yield (6.7 mg). ^{1}H NMR (500 MHz, CD_3OD): 7.30-7.03 (9H, m), 4.53 (1H, dd, *J* = 5.7, 9.1 Hz), 3.53 (1H, d, *J* = 14.5 Hz), 3.48 (1H, d, *J* = 14.5 Hz), 3.09 (1H, dd, *J* = 5.6, 13.9 Hz), 2.85 (1H, dd, *J* = 9.1, 13.9 Hz), 2.68 (3H, s). ^{13}C NMR (125 MHz, CD_3OD): 173.9, 173.8, 152.2, 152.2,

136.6, 134.2, 131.2, 130.1, 129.6, 127.9, 121.3, 121.3, 56.3, 43.6, 38.2, 26.3. ^{31}P NMR (CD_3OD): -3.8. HRMS (ESI) for $C_{18}H_{22}N_2O_6P$ [M + H$^+$] calculated 393.1215, obtained 393.1220.

(*S*)-4-(2-(2-(4-fluorophenyl)acetamido)-3-(methylamino)-3-oxopropyl)phenyl phosphate (5)

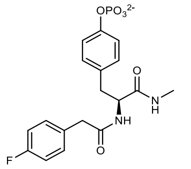

Compound **5** was obtained in 66% yield (8.9 mg). ^{1}H NMR (500 MHz, CD_3OD): 7.17-7.06 (6H, m), 6.98 (2H, m), 4.53 (1H, dd, *J* = 5.9, 8.9 Hz), 3.49 (1H, d, *J* = 14.6 Hz), 3.45 (1H, d, *J* = 14.6 Hz), 3.07 (1H, dd, *J* = 5.9, 13.8 Hz), 2.85 (1H, dd, *J* = 8.9, 13.8 Hz), 2.68 (3H, s). ^{13}C NMR (125 MHz, CD_3OD): 173.9, 173.6, 164.3, 162.3, 151.86, 151.80, 134.6, 132.56, 132.53, 131.90, 131.84, 131.32, 121.27, 121.23, 116.2, 116.0, 56.1, 42.6, 38.3, 26.3. ^{31}P NMR (CD_3OD): -3.7. HRMS (ESI) for $C_{18}H_{21}FN_2O_6P$ [M + H$^+$] calculated 411.1121, obtained 411.1120.

(*S*)-4-(2-(2-(4-methoxyphenyl)acetamido)-3-(methylamino)-3-oxopropyl)phenyl phosphate (6)

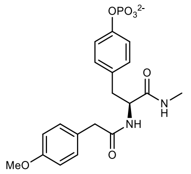

Compound **6** was obtained in 27% yield (3.8 mg). ^{1}H NMR (500 MHz, CD_3OD): 7.27 (2H, d, *J* = 8.0 Hz), 7.11 (4H, m), 7.06 (2H, d, *J* = 8.1 Hz), 4.58 (3H, s), 4.52 (1H, dd, *J* = 5.8, 8.9 Hz), 3.51 (1H, d, *J* = 14.4 Hz), 3.45 (1H, d, *J* = 14.4 Hz), 3.07 (1H, dd, *J* = 5.8, 13.8 Hz), 2.85 (1H, dd, *J* = 8.9, 13.8 Hz), 2.68 (3H, s). ^{13}C NMR (125 MHz, CD_3OD): 173.9, 173.8, 151.7, 141.4, 135.5, 134.6, 131.3, 130.1, 128.3, 121.2, 121.2, 64.9, 56.1, 43.4, 38.2, 26.3. ^{31}P NMR (CD_3OD): -3.7. HRMS (ESI) for $C_{19}H_{24}FN_2O_7P$ [M + H$^+$] calculated 423.1321, obtained 423.1321.

(*S*)-4-(2-(2-(4-(methoxycarbonyl)phenyl)acetamido)-3-(methylamino)-3-oxopropyl)phenyl phosphate (7)

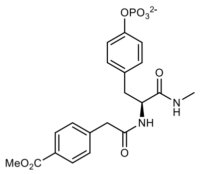

Compound **7** was obtained in 50% yield (7.4 mg). ^{1}H NMR (500 MHz, CD_3OD): 7.92 (2H, d, *J* = 8.2 Hz), 7.25 (2H, d, *J* = 8.2 Hz), 7.14 (2H, d, *J* = 8.3 Hz), 7.08 (2H, d, *J* = 8.3 Hz), 4.54 (1H, dd, *J* = 6.1, 8.9 Hz), 3.89 (3H, s), 3.57 (2H, s), 3.08 (1H, dd, *J* = 6.1, 13.8 Hz), 2.85 (1H, dd, *J* = 8.9, 13.8 Hz), 2.68 (3H, s). ^{13}C NMR (125 MHz, CD_3OD): 173.9, 172.9, 168.5, 151.9, 151.8, 142.3, 134.5, 131.3, 130.7, 130.4, 129.9, 121.3, 121.3, 56.2, 52.6, 43.4, 38.3, 26.3. ^{31}P NMR (CD_3OD): -3.6. HRMS (ESI) for $C_{20}H_{24}FN_2O_8P$ [M + H$^+$] calculated 451.1270, obtained 451.1264.

(*S*)-4-(3-(methylamino)-3-oxo-2-(2-(pyridin-4-yl)acetamido)propyl)phenyl phosphate (8)

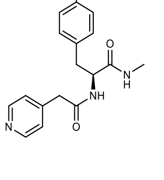

Compound **8** was obtained in 21% yield (2.7 mg). ^{1}H NMR (500 MHz, CD_3OD): 8.73 (2H, d, *J* = 6.5 Hz), 7.79 (2H, d, *J* = 6.6 Hz), 7.21 (2H, d, *J* = 8.4 Hz), 7.08 (2H, d, *J* = 8.4 Hz), 4.61 (1H, dd, *J* = 5.3, 10.1 Hz), 3.86 (2H, s), 3.16 (1H, dd, *J* = 5.3, 13.9 Hz), 2.84 (1H, dd, *J* = 10.1, 13.9 Hz), 2.72 (3H, s). ^{31}P NMR (CD_3OD): -3.7. MS (ESI) for $C_{17}H_{21}N_3O_6P$ [M + H$^+$] calculated 394.1, obtained 394.1.

Synthesis of the second-round library of SOCS2 ligands

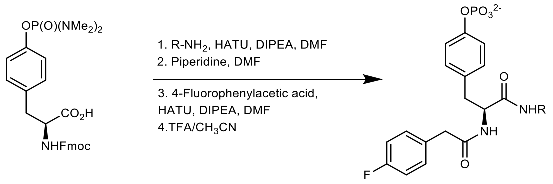

General Procedure A

Step 1. To a solution of Fmoc-Tyr(PO(NMe_2)$_2$)-OH (1 eq.) in DMF (10 mL/mmol) were added HATU (1 eq.) and the pH of the reaction mixture was adjusted to > 9 by addition of DIPEA (2-3 eq.). The resulting solution was stirred at rt for 5 min and then an amine (1.2 eq.) was added. The mixture was stirred for 1 h (until no presence of the starting materials was detected by LC-MS) and purified by reversed phase chromatography (20-95% CH_3CN in 0.1% aq. HCO_2H) to afford Fmoc-Tyr(PO(NMe_2)$_2$)-OR

Step 2. Fmoc deprotection was performed using 20% piperidine in DCM (10 mL/mmol). After solvent evaporation the residue was purified by column chromatography using Biotage KP-NH columns, elution gradient of 0 to 10% of MeOH in DCM, to afford H-Tyr(PO(NMe_2)$_2$)-OR

Step 3. H-Tyr(PO(NMe_2)$_2$)-OR (1eq) was added to a solution of 4-fluorophenylacetic acid (1 eq.), HATU (1 eq.) and DIPEA (2 eq.) in DMF and the reaction mixture was stirred for 1-2 h until no presence of the starting materials was detected by LC-MS. The mixture was purified by reversed phase chromatography (20-95% CH_3CN in 0.1% aq. HCO_2H) to afford 4-FC_6H_4-Tyr(PO(NMe_2)$_2$)-OR.

Step 4. 4-FC_6H_4-Tyr(PO(NMe_2)$_2$)-OR was dissolved in CH_3CN/TFA (0.3/0.3 mL) and stirred overnight. After solvent evaporation, the residue was purified by reversed phase chromatography (10-95% CH_3CN in 0.1% aq. HCO_2H) to afford the desired phosphotyrosine.

(*S*)-4-(3-((4-fluorobenzyl)amino)-2-(2-(4-fluorophenyl)acetamido)-3-oxopropyl)phenyl phosphate (9)

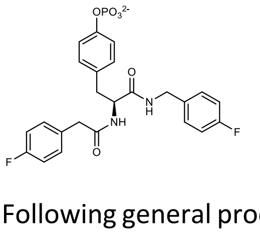

Following general procedure A, compound **9** was obtained using 4-fluorobenzylamine in 30% yield (7.4 mg). ^{1}H NMR (500 MHz, CD_3OD): 7.18-7.09 (8H, m), 7.03-6.96 (4H, m), 4.57 (1H, dd, *J* = 6.7, 8.3 Hz), 4.28 (2H, s), 3.48 (2H, m), 3.05 (1H, dd, *J* = 6.7, 13.7 Hz), 2.89 (1H, dd, *J* = 8.3, 13.7 Hz). ^{13}C NMR (125 MHz, CD_3OD): 173.6, 173.4, 164.4, 164.3, 162.5, 162.3, 152.91, 152.86, 135.59, 135.56, 133.3, 132.63, 132.61, 131.92, 131.86, 131.4, 131.2, 130.4, 130.3, 121.41, 121.37, 116.24, 116.17, 116.1, 116.0, 64.3, 56.4, 43.3, 42.5, 38.2. MS (ESI) for $C_{24}H_{24}F_2N_2O_6P$ [M + H$^+$] calculated 505.1, obtained 505.1

(*S*)-4-(2-(2-(4-fluorophenyl)acetamido)-3-((3-methylbenzyl)amino)-3-oxopropyl)phenyl phosphate (10)

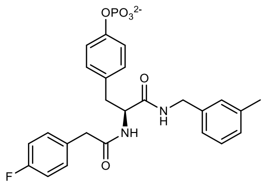

Following general procedure A, compound **10** was obtained using 3-methylbenzylamine in 21% yield (5.2 mg). ^{1}H NMR (500 MHz, DMSO-*d$_6$*): 8.45 (1H, t, *J* = 5.9 Hz), 8.33 (1H, d, *J* = 8.4 Hz), 7.20-6.92 (12H, m), 4.50 (1H, m), 4.22 (2H, m), 3.40 (2H, m), 2.96 (1H, m), 2.76 (1H, m), 2.25 (3H, s). ^{13}C NMR (125 MHz, DMSO-*d$_6$*): 171.2, 170.0, 162.0, 160.0, 150.7, 139.1, 137.4, 132.7, 132.5, 130.82, 130.76, 130.1, 128.3, 127.9, 127.4, 124.3, 119.7, 119.6, 114.9, 114.8, 54.3, 42.1, 41.1, 37.1, 21.1. MS (ESI) for $C_{25}H_{27}FN_2O_6P$ [M + H$^+$] calculated 501.1, obtained 501.1

(*S*)-4-(3-((4-fluoro-3-methylbenzyl)amino)-2-(2-(4-fluorophenyl)acetamido)-3-oxopropyl)phenyl phosphate (11)

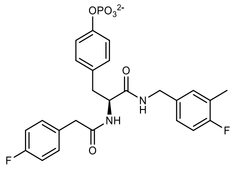

Following general procedure A, compound **11** was obtained using 4-fluoro-3-methylbenzylamine in 20% yield (5 mg). ^{1}H NMR (500 MHz, CD_3OD): 7.17-6.89 (11H, m), 4.57 (1H, dd, *J* = 6.4, 8.4 Hz), 4.24 (2H, m), 3.45 (2H, m), 3.06 (1H, dd, *J* = 6.4, 13.6 Hz), 2.89 (1H, dd, *J* = 8.4, 13.6 Hz), 2.21 (3H, d, *J* = 1.5 Hz). ^{13}C NMR (125 MHz, CD_3OD): 173.6, 173.3, 164.3, 162.8, 162.3, 160.9, 152.62, 152.57, 135.3, 135.2, 133.6, 132.63, 132.61, 131.9, 131.84, 131.82, 131.79, 131.2, 127.7, 127.6, 125.8, 125.7, 121.4, 121.3, 116.2, 116.1, 115.8, 115.7, 56.4, 43.4, 42.5, 38.2, 14.5, 14.4. MS (ESI) for $C_{25}H_{26}F_2N_2O_6P$ [M + H$^+$] calculated 519.1, obtained 519.1.

(*S*)-4-(3-((3-ethyl-4-fluorobenzyl)amino)-2-(2-(4-fluorophenyl)acetamido)-3-oxopropyl)phenyl phosphate (12)

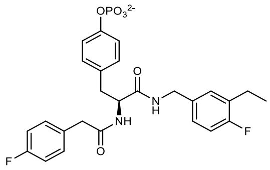

Following general procedure A, compound **12** was obtained using 3-ethyl-4-fluorobenzylamine in 28% yield (14.8 mg). ^{1}H NMR (500 MHz, CD_3OD): 7.20-7.05 (7H, m), 7.01-6.88 (4H, m), 4.60 (1H, dd, *J* = 6.5, 8.4 Hz), 4.26 (2H, s), 3.49 (1H, d, *J* = 14.7 Hz), 3.45 (1H, d, *J* = 14.7 Hz), 3.08 (1H, dd, *J* = 6.4, 13.8 Hz), 2.89 (1H, dd, *J* = 8.5, 13.8 Hz), 2.62 (2H, q, *J* = 7.6 Hz), 1.18 (3H, t, *J* = 7.6 Hz). ^{13}C NMR (125 MHz, CD_3OD): 173.6, 173.2, 164.3, 162.5, 162.3, 160.6, 151.87, 151.82, 135.44, 135.41, 134.4, 12.58, 132.56, 132.06, 131.94, 131.89, 131.82, 131.40, 130.40, 130.36, 127.76, 127.69, 121.29, 121.26, 116.2, 116.1, 115.9, 56.2, 43.5, 42.6, 40.5, 38.2, 23.12, 23.11, 14.9. ^{31}P NMR (CD_3OD): -5.1. ^{19}F NMR (CD_3OD): -118.3, -124.1. MS (ESI) for $C_{26}H_{28}F_2N_2O_6P$ [M + H$^+$] calculated 533.1, obtained 533.1.

(*S*)-4-(3-((3-allylbenzyl)amino)-2-(2-(4-fluorophenyl)acetamido)-3-oxopropyl)phenyl phosphate (14)

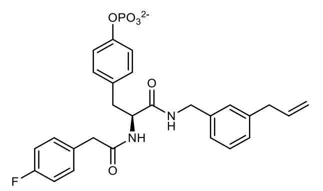

Following general procedure A, compound **14** was obtained using 3-allylbenzylamine (synthesized according to the literature procedure[2] in 27% yield (6.3 mg).

^{1}H NMR (500 MHz, CD_3OD): 7.26-6.93 (12H, m), 5.95 (1H, m), 5.09-4.99 (2H, m), 4.61 (1H, dd, *J* = 6.2, 8.8 Hz), 4.30 (2H, s), 3.49 (1H, d, *J* = 14.7 Hz), 3.45 (1H, d, *J* = 14.7 Hz), 3.34 (2H, d, *J* = 6.7 Hz), 3.10 (1H, dd, *J* = 6.2, 13.9 Hz), 2.89 (1H, dd, *J* = 8.9, 13.9 Hz). ^{31}P NMR (CD_3OD): -5.2. ^{19}F NMR (CD_3OD): -118.3. MS (ESI) for $C_{27}H_{29}FN_2O_6P$ [M + H$^+$] calculated 527.2, obtained 527.2.

(*S*)-4-(3-((3-allyl-4-fluorobenzyl)amino)-2-(2-(4-fluorophenyl)acetamido)-3-oxopropyl)phenyl phosphate (13)

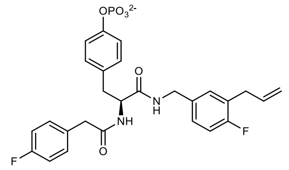

Following general procedure A, compound **13** was obtained using 3-allyl-4-fluorobenzylamine (synthesized according to the literature procedure in 11% yield (6.3 mg)[2]. ^{1}H NMR (500 MHz, CD_3OD): 7.18-7.04 (7H, m), 7.04-6.88 (4H, m), 5.93 (1H, m), 5.06-4.99 (2H, m), 4.59 (1H, dd, *J* = 6.3, 8.9 Hz), 4.26 (2H, s), 3.49 (1H, d, *J* = 14.9 Hz), 3.45 (1H, d, *J* = 14.9 Hz), 3.35 (2H, d, *J* = 6.3 Hz), 3.07 (1H, dd, *J* = 6.3, 13.8 Hz), 2.88 (1H, dd, *J* = 8.9, 13.9 Hz). ^{13}C NMR (125 MHz, CD_3OD): 173.6, 173.2, 164.3, 162.5, 162.3, 160.5, 152.0, 151.9, 137.1, 135.58, 135.55, 134.3, 132.59, 132.56, 131.59, 131.83, 131.4, 131.13, 131.09, 128.3,128.2, 121.31, 121.27, 116.4, 116.23, 116.19,116.06, 116.01, 56.2, 43.4, 12.6, 38.3, 33.96, 33.94. ^{31}P NMR (CD_3OD): -5.0. ^{19}F NMR (CD_3OD): -118.3, -123.2. MS (ESI) for $C_{27}H_{28}F_2N_2O_6P$ [M + H$^+$] calculated 545.1, obtained 545.1

Synthesis of MN551

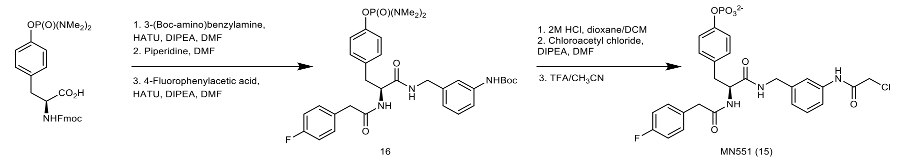

(*S*)-4-(3-((3-(2-chloroacetamido)benzyl)amino)-2-(2-(4-fluorophenyl)acetamido)-3-oxopropyl)phenyl phosphate (MN551)

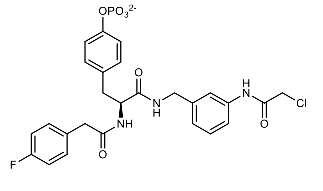

tert-butyl (*S*)-(3-((3-(4-((bis(dimethylamino)phosphoryl)oxy)phenyl)-2-(2-(4-fluorophenyl)-acetamido)propanamido)methyl)phenyl)carbamate **16** (14 mg, 0.021 mmol, synthesized according to general procedure A, Steps 1-3, using 3-(Boc-amino)benzylamine in 42% yield) was dissolved in a mixture of DCM (0.4 mL), 4M HCl in dioxane (0.4 mL), CH_3CN (0.2 mL) and stirred for 30 min (LCMS control). Volatile components were removed under high vacuum. The resulting amine hydrochloride

salt, which was used as crude without any further purification, was treated with chloroacetyl chloride (4 µL, 0.05 mmol), NEt_3 (15 µL, 0.1 mmol) in DMF (0.2 mL) for 1 h. The mixture was then concentrated *in vacuo*, and the crude was dissolved in CH_3CN/TFA (0.3/0.3 mL) and stirred overnight. After solvent evaporation, the residue was purified by reversed phase chromatography (10-95% CH_3CN in 0.1% aq. HCO_2H) to afford the desired compound **MN551** (5 mg, 41%). ^1H NMR (500 MHz, CD_3OD, mixture of rotamers): 8.36 (0.63H, m), 8.11 (0.37H, d, *J* = 7.6 Hz), 7.50 (1H, d, *J* = 7.9 Hz), 7.37 (1H, s), 7.26 (1H, t, *J* = 7.8 Hz), 7.19-7.04 (6H, m), 7.02-6.90 (3H, m), 4.57 (1H, dd, *J* = 6.5, 8.3 Hz), 4.32-4.27 (2H, m), 4.19 (2H, s), 3.55-3.43 (2H, m), 3.06 (1H, dd, *J* = 6.5, 13.8 Hz), 2.88 (1H, dd, *J* = 8.3, 13.8 Hz). ^{13}C NMR (125 MHz, CD_3OD): 171.2, 170.0, 164.7, 161.9, 160.0, 140.0, 138.5, 133.3, 132.5, 131.9, 130.83, 130.76, 130.2, 128.9, 122.8, 119.7, 119.6, 118.4, 118.2, 118.0, 114.9, 114.8, 54.2, 43.6, 42.2, 41.2. ^1P NMR (CD_3OD): -4.2. ^{19}F NMR (CD_3OD): -118.3. HRMS (ESI) for $C_{26}H_{27}ClFN_3O_7P$ [M + H$^+$] calculated 577.1259, obtained 577.1936.

Synthesis of MN551 prodrugs

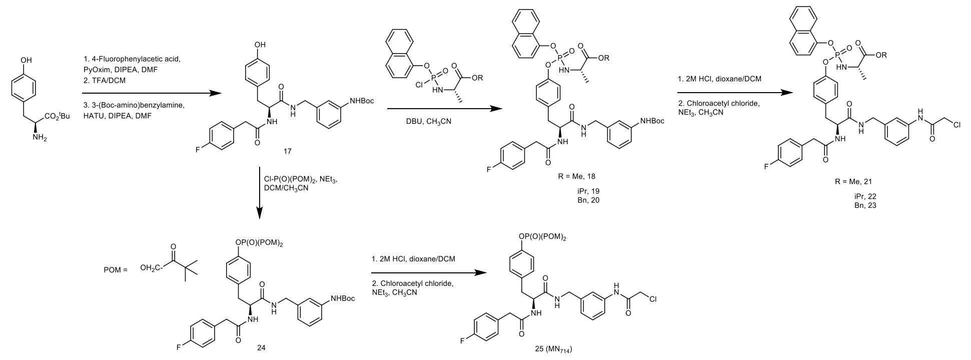

tert-butyl (*S*)-(3-((2-(2-(4-fluorophenyl)acetamido)-3-(4-hydroxyphenyl)propanamido)-methyl)phenyl)carbamate (17)

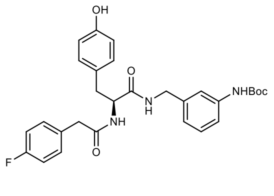

Step 1. H-Tyr-O^{t}Bu (474 mg, 2 mmol) was added to a solution of 4-fluorophenylacetic acid (304 mg, 2 mmol), PyOxim (1.05 g, 2 mmol) and DIPEA (0.8 mL, 4 mmol) in DMF (5 mL). The mixture was stirred for 1 h (LCMS control) and purified by reversed phase chromatography (20-95% CH_3CN in 0.1% aq. HCO_2H) to afford 4-FC_6H_4-Tyr-O^{t}Bu (589 mg, 79%), as a white solid.

Step 2. 4-FC_6H_4-Tyr-O^{t}Bu (589 mg, 1.58 mmol) was dissolved in a solution of 50% v/v trifluoroacetic acid in DCM. The resulting solution was stirred for 1 h or until complete conversion of starting material. Volatile components were removed, and the crude mixture was left under vacuum to remove any excess of TFA. The resulting carboxylic acid 4-FC_6H_4-Tyr-OH was used as crude in the next step without any further purification.

Step 3. To a solution of 4-FC_6H_4-Tyr-OH (1.58 mmol) in DMF (3 mL) were added HATU (600 mg, 1.58 mmol) and the pH of the reaction mixture was adjusted to > 9 by addition of DIPEA (3 eq.). The resulting solution was stirred at rt for 5 min and then 3-(Boc-amino)benzylamine (350 mg, 1.58 mmol) was added. The mixture was stirred for 1 h (LC-MS control) and purified by reversed phase chromatography (20-95% CH_3CN in 0.1% aq. HCO_2H) to afford **17** (680 mg, 83%) as a white solid.

^{1}H NMR (500 MHz, $CDCl_3$): 7.78 (1H, br), 7.23 (1H, d, *J* = 8.3 Hz), 7.11 (1H, t, *J* = 7.8 Hz), 7.06-6.78 (10H, m), 6.68 (1H, d, *J* = 7.6 Hz), 6.59 (1H, d, *J* = 8.3 Hz), 4.74 (1H, m), 4.21 (1H, dd, *J* = 5.7, 14.8 Hz), 4.06 (1H, dd, *J* = 4.9, 14.8 Hz), 3.37 (2H, m), 2.91 (2H, m), 2.18 (1H, s), 1.50 (9H, s). ^{13}C NMR (125 MHz, $CDCl_3$): 171.4, 171.3, 162.9, 160.9, 155.3, 153.2, 138.5, 138.2, 130.77, 130.71, 130.26, 130.20, 128.1, 127.4, 122.2, 118.2, 118.1, 115.62, 115.59, 115.4, 80.7, 57.8, 43.3, 42.2, 37.8, 28.3. ^{19}F NMR (CD_3OD): -115.2. MS (ESI) for $C_{29}H_{33}FN_3O_5$ [M + H$^+$] calculated 522.2, obtained 522.2.

(*S*)-4-(3-((3-(2-chloroacetamido)benzyl)amino)-2-(2-(4-fluorophenyl)acetamido)-3-oxopropyl)phenyl dipivaloyloxymethyl phosphate (MN714)

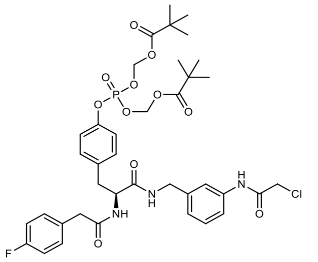

bis(POM) phosphoryl chloride (50 mg, 0.15 mmol) was added to a solution of **17** (50 mg, 0.1 mmol), NEt_3 (20 µL, 0.3 mmol), DMAP (13 mg, 0.1 mmol) in DCM (0.5 mL) and CH_3CN (0.5 mL), and the reaction mixture was stirred for 2 h (LCMS control)[3]. After solvent evaporation, the residue was purified by column chromatography (dry loading), elution gradient of 50 to 100% of EtOAc in heptane, to afford compound **24** (37 mg, 45%). Boc deprotection was achieved by treating the obtained product **24** (37 mg, 0.045 mmol) with a solution of 4M HCl in dioxane in DCM (50% v/v, 1 mL) for 15 min (LCMS control). Volatile components were removed, and the crude mixture was treated with chloroacetyl chloride (10 µL, 0.12 mmol), NEt_3 (30 µL, 0.2 mmol) in CH_3CN (0.5 mL) and DCM (0.5 mL) for 1 h. After solvent evaporation, the residue was purified by reversed phase chromatography (40-100% CH_3CN in 0.1% aq. HCO_2H) to afford the titled compound **MN714** (13 mg, 36%). ^{1}H NMR (500 MHz, $CDCl_3$): 8.59 (1H, s), 7.57 (1H, d, *J* = 8.1 Hz), 7.29 (1H, m), 7.20 (1H, br), 7.15-7.10 (2H, m), 7.08-6.93 (7H, m), 6.39 (1H, t, *J* = 5.6 Hz), 6.29 (1H, d, *J* = 7.7 Hz), 5.79-5.66 (4H, m), 4.61 (1H, dd, *J* = 7.7, 14.5 Hz), 4.31-4.21 (2H, m), 4.17 (2H, s), 3.47 (2H, s), 3.01 (1H, dd, *J* = 6.4, 13.7 Hz), 2.93 (1H, dd, *J* = 7.9, 13.7 Hz), 1.23 (18H, d, *J* = 3.0 Hz). ^{13}C NMR (125 MHz, $CDCl_3$): 176.65, 176.59, 170.9, 170.4, 164.3, 163.1, 161.2, 149.0, 148.97, 138.5, 137.5, 133.91, 133.9, 130.9, 130.8, 130.7, 130.1, 130.0, 129.4, 124.6, 120.3, 120.2, 119.52, 119.36, 115.92, 115.75, 83.14, 83.1, 83.05, 54.6, 43.5, 43.0, 42.5, 38.7, 37.6, 26.8. ^{31}P NMR (CD_3OD): -9.6. ^{19}F NMR (CD_3OD): -114.8. HRMS (ESI) for $C_{38}H_{47}ClFN_3O_{11}P$ [M + H$^+$] calculated 806.2621, obtained 806.2705.

Methyl ((4-((*S*)-3-((3-(2-chloroacetamido)benzyl)amino)-2-(2-(4-fluorophenyl)acetamido)-3-oxopropyl)phenoxy)(naphthalen-1-yloxy)phosphoryl)-L-alaninate (21)

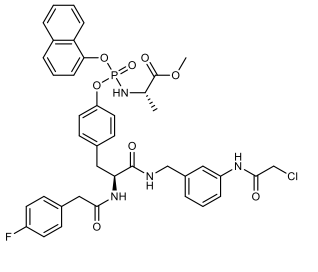

Methyl (chloro(naphthalen-1-yloxy)phosphoryl)-L-alaninate (30 mg, 0.09 mmol) was added to a solution of **17** (30 mg, 0.06 mmol), DBU (22 µL, 0.12 mmol), DMAP (8 mg, 0.06 mmol) in CH_3CN (0.3 mL) and the reaction mixture was stirred for 5 h (LCMS control)[4]. After solvent evaporation, the residue was purified by column chromatography (dry loading), elution gradient of 50 to 100% of EtOAc

in heptane, to afford compound **18** (21 mg, 43%). Boc deprotection was achieved by treating the obtained product **18** (21 mg, 0.026 mmol) with a solution of 4M HCl in dioxane in DCM (50% v/v, 1 mL) for 30 min. Volatile components were removed and the crude mixture was treated with chloroacetyl chloride (5 µL, 0.06 mmol), NEt_3 (15 µL, 0.1 mmol) in CH_3CN (0.2 mL) for 1 h. After solvent evaporation, the residue was purified by column chromatography, elution gradient of 0 to 20% of MeOH in DCM, to afford the titled compound **21** (16 mg, 78%). ^1H NMR (500 MHz, $CDCl_3$, mixture of two diastereoisomers): 8.74-8.65 (1H, m), 8.15-8.02 (1H, m), 7.89-7.83 (1H, m), 7.71-7.65 (1H, m), 7.58-7.47 (4H, m), 7.44-7.37 (1H, m), 7.24-6.85 (12H, m), 6.67-6.44 (2H, m), 4.69-4.57 (1H, m), 4.35-4.02 (6H, m), 3.64 (1.5H, s), 3.59 (1.5H, s), 3.41 (2H, m), 3.02-2.85 (2H, m), 1.39-1.33 (3H, m). ^{13}C NMR (125 MHz, $CDCl_3$, mixture of two diastereoisomers): 173. 7, 170.9, 170.8, 170.6, 170.5, 164.58, 164.42, 163.0, 161.1, 149.62, 149.57, 149.52, 149.47, 146.5, 138.45, 138.29, 137.5, 134.8, 133.47, 133.4, 130.83, 130.77, 130.6, 130.3, 130.1, 129.3, 127.9, 126.7, 126.5, 126.4, 125.51, 125.47, 125.1, 124.8, 124.6, 121.4, 120.39, 120.36, 120.32, 119.54, 119.47, 119.34, 115.8, 115.6, 115.1, 54.8, 54.7, 52.5, 52.4, 50.5, 50.4, 43.5, 43.1, 43.0, 42.5, 42.4, 38.1, 37.9, 20.94, 20.9, 20.86. ^{31}P NMR ($CDCl_3$, mixture of two diastereoisomers): -2.34, -2.38. ^{19}F NMR ($CDCl_3$, mixture of two diastereoisomers): -115.05, -115.08. HRMS (ESI) for $C_{40}H_{40}ClFN_4O_8P$ [M + H^+] calculated 789.2256, obtained 789.2396.

Isopropyl ((4-((*S*)-3-((3-(2-chloroacetamido)benzyl)amino)-2-(2-(4-fluorophenyl)acetamido)-3-oxopropyl)phenoxy)(naphthalen-1-yloxy)phosphoryl)-L-alaninate (22)

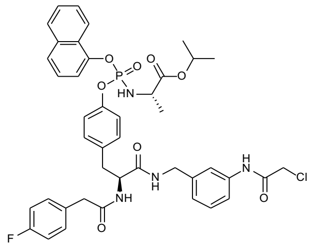

Prepared as reported above for **21**, starting from **17** and isopropyl (chloro(naphthalen-1-yloxy)phosphoryl)-L-alaninate[4]. Yield: 14 mg, 28% over 3 steps.

^1H NMR (500 MHz, $CDCl_3$, mixture of two diastereoisomers): 8.75-8.65 (1H, m), 8.14-8.02 (1H, m), 7.90-7.82 (1H, m), 7.73-7.64 (1H, m), 7.59-7.48 (4H, m), 7.44-7.37 (1H, m), 7.27-7.18 (2H, m), 7.15-7.04 (7H, m), 7.02-6.88 (7H, m), 6.47-6.28 (2H, m), 5.00-4.89 (1H, m), 4.66-4.53 (1H, m), 4.35-3.98 (9H, m), 3.81-3.75 (1H, m), 3.69-3.62 (1H, m), 3.47-3.38 (3H, m), 3.05-2.95 (2H, m), 2.93-2.83 (1H, m), 1.41-1.34 (3H, m), 1.25-1.14 (7H, m). ^{13}C NMR (125 MHz, $CDCl_3$, mixture of two diastereoisomers):172.7, 171.1, 170.9, 170.8, 170.6, 170.5, 170.4, 165.9, 164.5, 164.4, 163.1, 161.1, 149.7, 149.56, 149.5, 149.2, 146.6, 138.6, 138.4, 138.2, 137.5, 137.3, 134.8, 134.6, 133.43, 133.38, 130.84, 130.78, 130.6, 130.3, 130.1, 129.4, 129.3, 127.9, 126.7, 126.48, 126.4, 125.5, 125.1, 124.73, 124.66, 124.4, 121.4, 121.2, 120.43, 120.4, 119.6, 119.5, 119.4, 119.3, 119.1, 115.8, 115.7, 115.0, 72.3, 71.1, 69.49, 69.45, 61.7, 54.7, 54.3, 50.64, 50.57, 43.5, 43.4, 43.1, 43.0, 42.3, 40.9, 37.95, 37.9, 21.7, 21.5, 21.04, 21.0, 20.94. ^{31}P NMR ($CDCl_3$, mixture of two diastereoisomers): -2.26, -2.32. ^{19}F NMR ($CDCl_3$, mixture of two diastereoisomers): -115.02, -115.04. HRMS (ESI) for $C_{42}H_{44}ClFN_4O_8P$ [M + H^+] calculated 817.2569, obtained 817.2703.

Benzyl ((4-((*S*)-3-((3-(2-chloroacetamido)benzyl)amino)-2-(2-(4-fluorophenyl)acetamido)-3-oxopropyl)phenoxy)(naphthalen-1-yloxy)phosphoryl)-L-alaninate (23)

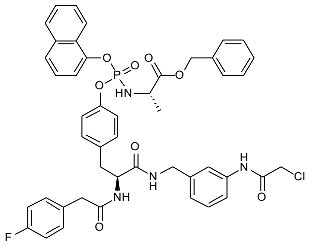

Prepared as reported above for **21**, starting from **17** and benzyl (chloro(naphthalen-1-yloxy)phosphoryl)-L-alaninate[4]. Yield: 10 mg, 19% over 3 steps.

^{1}H NMR (500 MHz, $CDCl_3$, mixture of two diastereoisomers): 8.74-8.60 (1H, m), 8.13-8.01 (1H, m), 7.90-7.82 (1H, m), 7.72-7.65 (1H, m), 7.60-7.45 (4H, m), 7.44-6.85 (19H, m), 6.50-6.30 (2H, m), 5.15-4.96 (2H, m), 4.67-4.51 (1H, m), 4.34-4.00 (7H, m), 3.46-3.41 (2H, m), 3.04-2.93 (1H, m), 2.93-2.82 (1H, m), 1.41-1.33 (3H, m). ^{13}C NMR (125 MHz, $CDCl_3$, mixture of two diastereoisomers): 173.04, 172.98, 170.89, 170.81, 170.5, 170.4, 164.5, 164.4, 163.1, 161.1, 149.5, 146.5, 138.4, 138.2, 137.5, 135.1, 134.8, 133.5, 133.4, 130.84, 130.78, 130.6, 130.3, 130.1, 129.3, 128.7, 128.6, 128.5, 128.13, 128.07, 127.9, 126.7, 126.5, 126.4, 125.5, 125.1, 124.7, 124.7, 124.6, 121.4, 121.2, 120.41, 120.38, 119.6, 119.5, 119.3, 115.8, 115.7, 115.2, 115.0, 67.4, 67.3, 54.8, 54.7, 54.4, 50.6, 50.5, 50.4, 43.5, 43.4, 43.1, 43.0, 42.5, 37.98, 37.93, 20.98, 20.94, 20.88. ^{31}P NMR ($CDCl_3$, mixture of two diastereoisomers): -2.42, -2.46. ^{19}F NMR ($CDCl_3$, mixture of two diastereoisomers): -115.00, -115.02. HRMS (ESI) for $C_{46}H_{44}ClFN_4O_8P$ [M + H$^+$] calculated 865.2569, obtained 865.2708.

Synthesis of the fluorescent probe 26

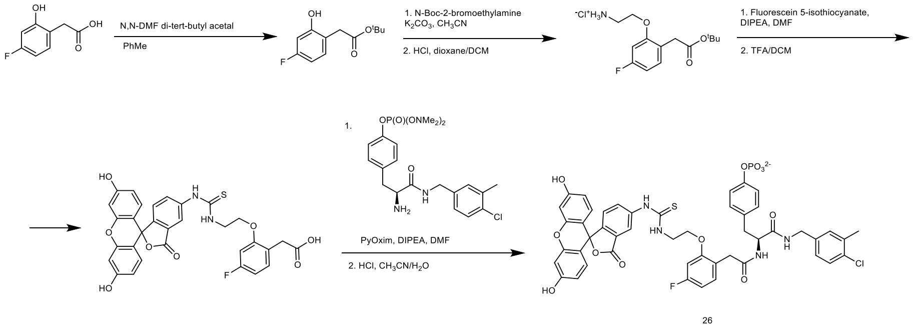

***Tert*-butyl 2-(4-fluoro-2-hydroxyphenyl)acetate**

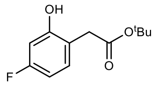

N,*N*-Dimethylformamide di-*tert*-butyl acetal (1.5 mL, 6 mmol) was added to a suspension of 2-(4-fluoro-2-hydroxyphenyl)acetic acid (340 mg, 2 mmol) in toluene (10 mL) and the reaction mixture was stirred at 80 °C for 2 h. Volatile components were removed, and the crude mixture was treated with water and extracted with EtOAc. Organic fractions were combined, dried over $MgSO_4$ and concentrated. The residue was purified by column chromatography, elution gradient of 0 to 100% of EtOAc in heptane, to afford the title compound (327 mg, 72%). ^{1}H NMR (500 MHz, $CDCl_3$): 8.32 (1H, s), 7.00 (1H, t, *J* = 7.4 Hz), 6.68 (1H, dd, *J* = 10.2, 2.5 Hz), 6.68 (1H, dt, *J* = 8.3, 2.5 Hz), 3.55, (2H, s), 1.47 (9H, s). ^{13}C NMR (125 MHz, $CDCl_3$) 173.7, 164.2, 162.2, 157.0, 156.9, 131.6, 131.5, 116.8, 116.8, 107.5, 107.3, 105.6, 105.4, 83.3, 39.3, 27.9.

2-(2-(2-(*tert*-butoxy)-2-oxoethyl)-5-fluorophenoxy)ethan-1-aminium chloride

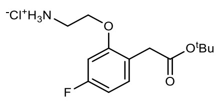

A mixture of *tert*-butyl 2-(4-fluoro-2-hydroxyphenyl)acetate (45 mg, 0.2 mmol), N-Boc-2-bromoethylamine (45 mg, 0.2 mmol) and K_2CO_3 (80 mg, 0.6 mmol) in acetonitrile (1 mL) was refluxed for 8 h. Volatile components were removed and the crude mixture was purified by column chromatography, elution gradient of 0 to 100% of EtOAc in heptane, to afford *tert*-butyl 2-(2-(2-((*tert*-butoxycarbonyl)amino)ethoxy)-4-fluorophenyl)acetate (42 mg, 60%). Boc deprotection was achieved by treating the obtained product with a solution of 4M HCl in dioxane in DCM (50% v/v, 1 mL) for 30 min. Volatile components were removed and the crude amine was used in the next step without any further purification. NMR (500 MHz, DMSO-d_6): 8.32 (3H, br s), 7.22 (1 H, m), 6.92 (1 H, m), 6.74 (1 H, m), 4.20 (2H, s), 4.62 (2H, m), 3.16(2H, m), 1.38 (9H, s). ^{13}C NMR (125 MHz, DMSO-d_6): 172.9, 163.0, 161.0, 157.0, 156.9, 132.0, 131.9, 120.3, 120.2, 107.1, 106.9, 100.3, 100.1, 80.1, 64.9, 38.3, 34.6, 27.8. MS (ESI) for $C_{14}H_{21}FNO_3$ [M + H$^+$] calculated 270.1, obtained 270.2.

2-(2-(2-(3-(3',6'-dihydroxy-3-oxo-3H-spiro[isobenzofuran-1,9'-xanthen]-5-yl)thioureido)ethoxy)-4-fluorophenyl)acetic acid

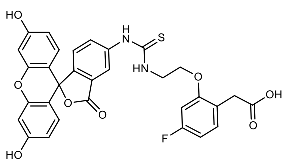

A mixture of fluorescein 5-isothiocyanate (10 mg, 0.025 mmol), *tert*-butyl 2-(2-(2-aminoethoxy)-4-fluorophenyl)acetate (6 mg, 0.022 mmol) and DIPEA (14 µL, 0.075 mmol) in DMF (0.2 mL) was stirred overnight at rt. The reaction mixture was injected directly to HPLC and purified by preparative HPLC under acidic conditions to give tert-butyl 2-(2-(2-(3-(3',6'-dihydroxy-3-oxo-3H-spiro[isobenzofuran-1,9'-xanthen]-5-yl)thioureido)ethoxy)-4-fluorophenyl)acetate, which was treated a solution of 50% v/v TFA in DCM for 1 h. Volatile components were removed, and the carboxylic acid was left under vacuum to remove any excess of TFA. The resulting title compound was used as crude in the next step without any further purification. MS (ESI) for $C_{31}H_{24}FN_2O_8S$ [M + H$^+$] calculated 603.1, obtained 603.2.

(*S*)-4-(3-((4-chloro-3-methylbenzyl)amino)-2-(2-(2-(2-(3-(3',6'-dihydroxy-3-oxo-3H-spiro[isobenzofuran-1,9'-xanthen]-5-yl)thioureido)ethoxy)-4-fluorophenyl)acetamido)-3-oxopropyl)phenyl dihydrogen phosphate (26)

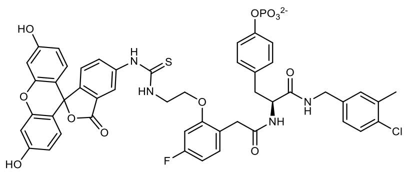

PyOxim (8 mg, 0.015 mmol) was added to a solution of 2-(2-(2-(3-(3',6'-dihydroxy-3-oxo-3H-spiro[isobenzofuran-1,9'-xanthen]-5-yl)thioureido)ethoxy)-4-fluorophenyl)acetic acid (9 mg, 0.015 mmol), (*S*)-4-(2-amino-3-((4-chloro-3-methylbenzyl)amino)-3-oxopropyl)phenyl bis((dimethylamino)oxy)phosphinate (7 mg, 0.015 mmol; synthesized according to general procedure A, Steps 1-2, using (4-chloro-3-methylphenyl)methanamine in 50% yield) and DIPEA (8 µL, 0.045mmol) in DMF (0.85 mL). The mixture was stirred for 1 h (LC-MS control) and was injected directly to preparative HPLC and purified under acidic conditions. The obtained intermediate was treated with 4M HCl in CH_3CN/water (0.2 mL/0.2 mL) and stirred overnight. After solvent evaporation, the residue

was purified by preparative HPLC under acidic conditions to afford the desired compound **26** (2 mg, 13%). ^{1}H NMR (500 MHz, CD_3OD): 8.24 (1H, br s), 7.69 (1H, br d, *J* = 6.8 Hz), 7.24-6.99 (10H, m), 6.90 (1H, m), 6.81 (1H, m), 6.73-6.52 (8H, m), 4.54 (1H, m), 4.22 (2H, s), 4.10 (2H, m), 3.91 (2H, br s), 3.52 (2H, m), 3.01 (1H, dd, *J* = 6.1, 13.8 Hz), 2.89 (1H, dd, *J* = 8.3, 13.8 Hz), 2.24 (3H, s). ^{31}P NMR (CD_3OD): -5.9. ^{19}F NMR (CD_3OD): -115.6. MS (ESI) for $C_{48}H_{42}ClFN_4O_{12}PS$ [M + H^+] calculated 983.1930, obtained 983.2028.

REFERENCES

1 Kung, W. W., Ramachandran, S., Makukhin, N., Bruno, E. & Ciulli, A. Structural insights into substrate recognition by the SOCS2 E3 ubiquitin ligase. *Nat Commun* **10**, 2534, doi:10.1038/s41467-019-10190-4 (2019).

2 Duffy, K. J. *et al.* Chemical Compounds. WO/2019/053617 (2019).

3 Hwang, Y. & Cole, P. A. Efficient synthesis of phosphorylated prodrugs with bis(POM)-phosphoryl chloride. *Org Lett* **6**, 1555-1556, doi:10.1021/ol049714v (2004).

4 Miccoli, A., Dhiani, B. A. & Mehellou, Y. Phosphotyrosine prodrugs: design, synthesis and anti-STAT3 activity of ISS-610 aryloxy triester phosphoramidate prodrugs. *MedChemComm* **10**, 200-208, doi:10.1039/c8md00244d (2019).
