# Supplementary material for: Structure-based design of a phosphotyrosine-masked covalent ligand targeting the E3 ligase SOCS2
Source: Nat Commun. 2023 Oct 10;14:6345. doi: 10.1038/s41467-023-41894-3 (PMC10564737; doi:10.1038/s41467-023-41894-3)
Supplement: Supplementary file 2 — Reporting Summary [file 41467_2023_41894_MOESM2_ESM.pdf]

nature portfolio

Corresponding author(s): Alessio Ciulli
Last updated by author(s): Sep 2, 2023

# Reporting Summary

Nature Portfolio wishes to improve the reproducibility of the work that we publish. This form provides structure for consistency and transparency in reporting. For further information on Nature Portfolio policies, see our Editorial Policies and the Editorial Policy Checklist.

## Statistics

For all statistical analyses, confirm that the following items are present in the figure legend, table legend, main text, or Methods section.

| n/a | Confirmed | |
|---|---|---|
| ☐ | ☒ | The exact sample size (*n*) for each experimental group/condition, given as a discrete number and unit of measurement |
| ☐ | ☒ | A statement on whether measurements were taken from distinct samples or whether the same sample was measured repeatedly |
| ☒ | ☐ | The statistical test(s) used AND whether they are one- or two-sided<br>*Only common tests should be described solely by name; describe more complex techniques in the Methods section.* |
| ☒ | ☐ | A description of all covariates tested |
| ☒ | ☐ | A description of any assumptions or corrections, such as tests of normality and adjustment for multiple comparisons |
| ☐ | ☒ | A full description of the statistical parameters including central tendency (e.g. means) or other basic estimates (e.g. regression coefficient) AND variation (e.g. standard deviation) or associated estimates of uncertainty (e.g. confidence intervals) |
| ☒ | ☐ | For null hypothesis testing, the test statistic (e.g. *F*, *t*, *r*) with confidence intervals, effect sizes, degrees of freedom and *P* value noted<br>*Give P values as exact values whenever suitable.* |
| ☒ | ☐ | For Bayesian analysis, information on the choice of priors and Markov chain Monte Carlo settings |
| ☒ | ☐ | For hierarchical and complex designs, identification of the appropriate level for tests and full reporting of outcomes |
| ☒ | ☐ | Estimates of effect sizes (e.g. Cohen's *d*, Pearson's *r*), indicating how they were calculated |

*Our web collection on statistics for biologists contains articles on many of the points above.*

## Software and code

Policy information about availability of computer code

| | |
|---|---|
| Data collection | X-ray diffraction data: Diffraction data were collected at Diamond Light Source beamline i03/i04 or The European Synchrotron Radiation Facility ID30-A1<br>Western blotting/SDS gels: ChemiDoc Touch imaging system (BioRad) operated by Image Lab (v2.4.0.03).<br>19F CPMG NMR spectroscopy: Avance III 500 MHz Bruker spectrometer equipped with a 5mm CPQCI 1H/19F/13C/15N/D Z-GRD cryoprobe<br>Fluorescence polarization assays: BMG Labtech PHERAstar (firmware v1.33)<br>SPR: Biacore S200 instrument (GE Healthcare)<br>ITC: ITC200 instrument (Malvern)<br>CETSA: GloMax® Discover System (software v4.0.0, firmware v4.92)<br>In cell NMR: Bruker AVANCE III spectrometer with an 11.7 T magnet using a QCI-F cryoprobe at 470 MHz Lamour frequency<br>LC-MS/MS : Q Exactive™ plus, Mass Spectrometer (Thermo Scientific) coupled to a Dionex Ultimate 3000 RS (Thermo Scientific) |
| Data analysis | X-ray structure solution: Diffraction images were processed with Xia2 Dials/ autoPROC. The structure was solved by molecular replacement using SBC crystal structure (PDB entry 2C9W) as a search model. Subsequent iterative model building, and refinement was done according to standard protocols using COOT and Phenix. Ligand restraints were generated using the Phenix eLBOW/PRODRG server.<br>19F CPMG NMR spectroscopy: All NMR data were processed and analysed using TopSpin (Bruker).<br>SPR: Biacore Evaluation Software (GE Healthcare).<br>Fluorescence polarization assays: GraphPad Prism 9<br>ITC:  MicroCal PEAQ-ITC Analysis Software1.1.0.1262 and MicroCal ITC-ORIGIN Analysis Software 7.0 (Malvern)<br>CETSA: Data fitted to obtain apparent Tagg values using the Boltzmann Sigmoid equation using Graphpad Prism 9<br>In cell NMR: Topspin 4.1.1 (Bruker). |

LC-MS/MS : MaxQuant software
Western blot imaging: Image Lab (Bio-Rad)

For manuscripts utilizing custom algorithms or software that are central to the research but not yet described in published literature, software must be made available to editors and reviewers. We strongly encourage code deposition in a community repository (e.g. GitHub). See the Nature Portfolio guidelines for submitting code & software for further information.

## Data

Policy information about availability of data

All manuscripts must include a data availability statement. This statement should provide the following information, where applicable:
- Accession codes, unique identifiers, or web links for publicly available datasets
- A description of any restrictions on data availability
- For clinical datasets or third party data, please ensure that the statement adheres to our policy

X-ray crystallography data have been deposited to the PDB under accession codes 7ZLP (compound 9 soaked in complex with SBC), 7ZLN (compound 11 soaked in complex with SBC), 7ZLO (compound 12 soaked in complex with SBC), 7ZLR (compound 13 soaked in complex with SBC), 7ZLS (compound 13 co-crystallized in complex with SBC), 7ZLM (compound MN551 co-crystallized in complex with SBC). NMR spectra for MN714 is provided in the Supplementary Information. All other data generated for all Tables, Figures and Supplementary Figures are available in the Supplementary Data files. Plasmids generated in this study are available from the corresponding authors upon request. Source data are provided with this paper.

## Human research participants

Policy information about studies involving human research participants and Sex and Gender in Research.

| | |
|---|---|
| Reporting on sex and gender | Not applicable |
| Population characteristics | Not applicable |
| Recruitment | Not applicable |
| Ethics oversight | Not applicable |

Note that full information on the approval of the study protocol must also be provided in the manuscript.

# Field-specific reporting

Please select the one below that is the best fit for your research. If you are not sure, read the appropriate sections before making your selection.

☒ Life sciences ☐ Behavioural & social sciences ☐ Ecological, evolutionary & environmental sciences

For a reference copy of the document with all sections, see nature.com/documents/nr-reporting-summary-flat.pdf

# Life sciences study design

All studies must disclose on these points even when the disclosure is negative.

| | |
|---|---|
| Sample size | Sample sizes were not predetermined using statistical analyses. Sample sizes were based on prior experience in the field and our previous studies (Zengerle et al. ACS Chem Biol 2015; Gadd et al. Nat. Chem. Biol. 2017; Kung et al. Nat Commun. 2019; Imaide et al. Nat Chem Biol. 2021). |
| Data exclusions | No data excluded |
| Replication | Unless stated in figure legends or method sections, all experiments were done at least twice and the reproduction were successful. The number of technical replicates and independent biological experiments are specified in the respective figure legends. |
| Randomization | There was no requirement for randomization of data. |
| Blinding | No blinding was performed, as no subjective measurements were done. |

# Reporting for specific materials, systems and methods

We require information from authors about some types of materials, experimental systems and methods used in many studies. Here, indicate whether each material, system or method listed is relevant to your study. If you are not sure if a list item applies to your research, read the appropriate section before selecting a response.

## Materials & experimental systems

| n/a | Involved in the study | |
|---|---|---|
| ☐ | ☒ | Antibodies |
| ☐ | ☒ | Eukaryotic cell lines |
| ☒ | ☐ | Palaeontology and archaeology |
| ☒ | ☐ | Animals and other organisms |
| ☒ | ☐ | Clinical data |
| ☒ | ☐ | Dual use research of concern |

## Methods

| n/a | Involved in the study | |
|---|---|---|
| ☒ | ☐ | ChIP-seq |
| ☒ | ☐ | Flow cytometry |
| ☒ | ☐ | MRI-based neuroimaging |

# Antibodies

| | |
|---|---|
| Antibodies used | The following primary antibodies were used for immunoblot analyses: SOCS2 (no. ab109245, Abcam, 1:1000 dilution),<br><br>The following secondary antibodies used were IRDye® 800CW anti-rabbit (no. 926-32211, LiCor, 1:10000 dilution), and hFABTM rhodamine anti-tubulin (no. 12004165, Biorad, 1:5000 dilution). |
| Validation | Target specificity for the following antibodies were previously confirmed by the Ciulli group:<br>- anti-SOCS2 (no. ab109245, Abcam): Knockdown validated, disappearance of the band in immunoblotting siRNA treatment<br>Validation with siRNA provided in source file |

# Eukaryotic cell lines

Policy information about cell lines and Sex and Gender in Research

| | |
|---|---|
| Cell line source(s) | K562 and HeLa cells were obtained from ATCC. |
| Authentication | Cell lines were authenticated by vendors and routinely authenticated by visual inspection of cell morphology |
| Mycoplasma contamination | All used cell lines were routinely tested and confirmed negative for mycoplasma contamination. |
| Commonly misidentified lines<br>(See ICLAC register) | No commonly misidentified cell lines were used. |
